# Socioeconomic factors impacting treatment delays in oral and oropharyngeal squamous cell carcinoma: a systematic review

**DOI:** 10.1590/0102-311XEN121324

**Published:** 2025-04-11

**Authors:** Débora Rosana Alves Braga Silva Montagnoli, Vitória Ferreira Leite, Yasmim Silva Godoy, Carolina Castro Martins-Pfeifer, Johana Alejandra Moreno-Drada, Maria Cássia Ferreira Aguiar, Mauro Henrique Nogueira Guimarães Abreu, Renata de Castro Martins

**Affiliations:** 1 Universidade Federal de Minas Gerais, Belo Horizonte, Brasil.; 2 Universidad del Valle, Cali, Colombia.

**Keywords:** Oral Cancer, Oropharyngeal Cancer, Squamous Cell Carcinoma, Treatment Delay, Socioeconomic Factors, Câncer Oral, Câncer Orofaríngeo, Carcinoma de Células Escamosas, Atraso no Tratamento, Fatores Socioeconômicos, Cáncer Oral, Cáncer Orofaríngeo, Carcinoma de Células Escamosas, Retraso del Tratamiento, Factores Socioeconómicos

## Abstract

This systematic review investigates associations between socioeconomic factors and treatment delay in adults with oral and oropharyngeal squamous cell carcinoma (OOSCC). Observational studies were searched across five databases (PubMed, Scopus, Web of Science, Embase, and Virtual Health Library) and grey literature. No restrictions were imposed about language or year of publication. Risk of bias was analyzed using the Joanna Briggs Institute tool. The primary outcome was defined as the cut-off points of treatment delays, which was addressed by the studies included. The certainty of evidence was assessed following the *Grading of Recommendations Assessment, Development, and Evaluation* (GRADE) approach. In total, 10 retrospective cohort studies were included in the narrative synthesis. Type of insurance plan, sex, older ages, non-white patients, low education level, treatment in public or regional hospital, need for transition care, and living in regional/remote areas were factors associated with treatment delay for OOSCC. However, methodological limitations regarding the adjustment for confounders, the heterogeneity of the definition of delay (different cut-off points), socioeconomic variables, and the lack of eligible articles from different countries resulted in a very low certainty of evidence due to severe issues of bias, inconsistency, and indirectness according to the GRADE guidelines. The association between socioeconomic factors and OOSCC treatment delay is inconclusive by the available data. Given the complexity of the determinants of access to timely OOSCC treatment, further research is recommended in different countries.

## Abstract (pt)

Esta revisão sistemática investiga associações entre fatores socioeconômicos e atraso no tratamento em adultos com carcinoma espinocelular (CEC) de boca e orofaringe. Estudos observacionais foram pesquisados em cinco bases de dados (PubMed, Scopus, Web of Science, Embase e Biblioteca Virtual em Saúde) e literatura cinzenta. Não houve restrição sobre idioma ou ano de publicação. O risco de viés foi avaliado usando a ferramenta do Joanna Briggs Institute. Os pontos de corte representando atraso no tratamento, abordado pelos estudos incluídos, foram definidos como desfecho primário. A certeza das evidências foi avaliada usando a abordagem *Grading of Recommendations Assessment, Development, and Evaluation* (GRADE). Ao todo, dez estudos de coorte retrospectivo foram incluídos na síntese narrativa. Tipo de plano de saúde, sexo, idade avançada, pacientes não brancos, baixo nível de escolaridade, tratamento em hospital público/regional, necessidade de cuidados de transição e residir em áreas isoladas/remotas foram fatores associados ao atraso no tratamento do CEC. No entanto, as limitações metodológicas para o ajuste para fatores de confusão, a heterogeneidade da definição de atraso (diferentes pontos de corte) e das variáveis socioeconômicas abordadas e a falta de artigos elegíveis de diferentes países resultaram em uma evidência com nível de certeza muito baixo devido a graves riscos de viés, inconsistência e evidência indireta de acordo com o GRADE. Com base nos dados disponíveis, a associação entre fatores socioeconômicos e atraso no tratamento do CEC é inconclusiva. Dada a complexidade dos determinantes do acesso ao tratamento oportuno do CEC, recomendamos mais pesquisas em diferentes países.

## Abstract (es)

Esta revisión sistemática evalúa las asociaciones entre los factores socioeconómicos y el tratamiento tardío en adultos con carcinoma de células escamosas (CCE) oral y orofaríngeo. Se realizaron búsquedas de estudios observacionales en cinco bases de datos (PubMed, Scopus, Web of Science, Embase y Biblioteca Virtual en Salud) y en la literatura gris. No se impusieron restricciones sobre el idioma o el año de publicación. El riesgo de sesgo se evaluó utilizando la herramienta del Instituto Joanna Briggs. Los puntos de cohorte que representan el tratamiento tardío abordado por los estudios incluidos se definieron como el resultado primario. La certeza de la evidencia se evaluó utilizando el enfoque *Grading of Recommendations Assessment, Development, and Evaluation* (GRADE). En total, se incluyeron diez estudios de cohorte retrospectivos en la síntesis narrativa. El tipo de plan de salud, el sexo, la edad avanzada, los pacientes no blancos, el bajo nivel de educación, el tratamiento en un hospital público/regional, la necesidad de atención transitoria y la residencia en zonas aisladas/remotas fueron los factores asociados con el tratamiento tardío del CCE. Sin embargo, las limitaciones metodológicas para ajustar los factores de confusión, la heterogeneidad de la definición de retraso (diferentes puntos de cohorte) y de las variables socioeconómicas abordadas, y la falta de artículos elegibles de diferentes países dieron como resultado una evidencia con nivel muy bajo de certeza debido a los graves riesgos de sesgo, inconsistencia y evidencia indirecta de acuerdo con GRADE. La asociación entre los factores socioeconómicos y retraso en el tratamiento del CCE no es concluyente según los datos disponibles. Dada la complejidad de los determinantes del acceso al tratamiento oportuno del CCE, se recomienda más estudios de diferentes países.

## Introduction

A socioeconomic gradient is found in oral health issues, especially oral and oropharyngeal squamous cell carcinoma (OOSCC) ^1^ ^,^ ^2^. Higher incidences of OOSCC are reported in patients with low socioeconomic status in contexts of self-neglect and difficulty accessing preventive healthcare ^1^ ^,^ ^2^. Inequalities regarding treatment for OOSCC are related to the difficulty in accessing health services, especially among more vulnerable populations. Poorer individuals often have more advanced stages of the condition and, consequently, a worse prognosis and higher mortality rate ^3^ ^,^ ^4^.

Besides socioeconomic factors, the time elapsed between diagnosis and the onset of treatment is an important aspect in cases of OOSCC. The *Aarhus Statement* was proposed to standardize time points and intervals in screening for early cancer detection ^5^. The declaration suggests four key time points: date of first symptom, date of first presentation, date of referral, and date of diagnosis. Güneri & Epstein ^6^ established three main types of delay in cases of oral cancer: “patient delay”, which refers to the time from the first detection of a sign/symptom to the patient seeking care; “professional delay”, which is the time from the first examination/assessment by a healthcare professional to the final histological diagnosis of the malignancy; and “system delay”, which is the time from the final diagnosis to the onset of the first intervention. While the first two allude to diagnosis delay, the latter is related to treatment delay and involves broader structural issues, including access to healthcare.

Treatment delay has been reported as a consequence of diagnosis delay ^7^. However, the causes of treatment delay or system delay in cases of OOSCC need to be better understood. In addition to the characteristics of the disease, micro- and macro-social factors need to be considered ^5^ ^,^ ^8^. Access to treatment is linked to the use of health services, which involves individual and contextual determinants ^9^. Delayed cancer treatment is an issue that impacts health systems worldwide ^10^. Ideally, patients should begin treatment as soon as they are diagnosed. As this is often not possible, studies have shown that up to 30 days to the onset of treatment is considered acceptable for a satisfactory prognosis and greater chance of survival in patients with OOSCC ^11^ ^,^ ^12^ ^,^ ^13^ ^,^ ^14^. The impact of socioeconomic disparities on treatment delay for various types of cancer, such as breast cancer ^15^, colorectal cancer ^16^, hepatocellular carcinoma ^17^, and brain cancer ^18^, has been demonstrated. The evidence is rarer for OOSCC, but we can presume that this type of cancer is not the exception. It is important to investigate the association between social determinants of health related to the population’s living conditions and the provision of health services. Thus, this systematic review aimed to investigate associations between socioeconomic factors and treatment delay in patients with OOSCC.

## Material and methods

### Eligibility criteria

This review was reported following the *Preferred Reporting Items for Systematic Reviews and Meta-Analyses* (PRISMA statement, 2020) ^19^. The research question was: “Is there an association between socioeconomic factors and treatment delay in cases of OOSCC?”. The question was formed considering the PECO framework for observational studies ^6^:

The inclusion criteria were patients with oral cancer or tumors in specific sites of the oral cavity and oropharynx; adults (≥ 18 years of age); studies with a histological diagnosis of squamous cell carcinoma; any socioeconomic factor; studies that assessed the time from diagnosis to the onset of treatment; and observational studies (cross-sectional, cohort, and case-control studies). No restrictions were imposed regarding language or year of publication.

Studies that reported head and neck cancers without considering oral and oropharyngeal cancers separately from other cancers (e.g., skin, lymph node, and thyroid cancers); and neoplasms of histological types other than squamous cell carcinoma, as they may show different progression rates and symptoms ^20^, which may exert an influence on treatment delay were excluded.

### Search strategy

In total, five databases were searched from inception to January 2023, updated in December 2023: MEDLINE (PubMed), Embase (Ovid), Scopus, Web of Science, and Virtual Health Library (BIREME). Grey literature was searched via OpenGrey and Google Scholar (the latter limited to the first 100 results listed). Specific search strategies were developed for each database and reviewed by an expert in systematic reviews. The reference lists of the articles included in the review were hand searched in an attempt to find additional relevant articles. The Box S1 (Supplementary Material; https://cadernos.ensp.fiocruz.br/static//arquivo/suppl-e00121324_8794.pdf) details the employed search strategies.

### Selection process

The articles retrieved from the databases were exported to EndNote Web (http://myendnoteweb.com) and duplicates were removed. Titles and abstracts were independently screened by two pairs of reviewers (D.R.A.B.S.M./V.F.L. and D.R.A.B.S.M./Y.S.G.). The Rayyan software (https://www.rayyan.ai/) was used for the screening process ^21^. Articles that met the inclusion criteria were selected for full-text analysis, which was performed by the same reviewers. Divergences across stages were resolved by discussion until consensus. If disagreement persisted, a third reviewer (R.C.M.) made the final decision. Prior to each phase, the main investigator trained the reviewers with a sample of 10% of the articles.

### Data extraction

A Microsoft Excel spreadsheet (https://products.office.com/) was created for data extraction. The form was tested with a sample of four articles. Data were independently extracted by one pair of reviewers (D.R.A.B.S.M./V.F.L.) and a consensus was reached via discussion. The senior author cross-checked all data.

The following data were extracted from each study: authors, year of publication, country, design, sample size, sex distribution, age group, definition of treatment delay, distribution of each socioeconomic variable, association measures, funding, conflicts of interest, and conclusion.

### Assessment of risk of bias

The risk of bias was assessed by two independent reviewers (D.R.A.B.S.M. and C.C.M.P.) using the Joanna Briggs Institute (JBI) *Critical Checklist for Cohort Studies* ^22^. This tool is composed of 11 domains, each of which is rated as “yes”, “no”, “not applicable”, or “unclear”. Inclusion criteria were recruiting similar groups from the same population, measuring exposures, presenting confounders and strategies for dealing with them, showing groups/participants without the outcome at baseline (or at the time of exposure), measuring outcomes, reporting a complete follow-up time, presenting strategies for dealing with incomplete follow-up, and showing an appropriate statistical analysis. Sex and age were considered required confounders selected for the adjusted analysis. The reviewers had previously undergone training and calibration exercises coordinated by a researcher with expertise in assessing the quality of observational studies (C.C.M.P.). Divergences of opinion were discussed until a consensus was reached.

### Data items

The primary outcome of treatment delay was defined according to the cut-off points of the longest time between diagnosis and treatment addressed by the studies included: ≥ 30 days ^11^ ^,^ ^13^ ^,^ ^23^, ≥ 38 days ^24^, ≥ 45 days ^12^, ≥ 50 days ^25^, and > 6 weeks ^26^.

The socioeconomic variables were selected according to factors reported in the articles. Thus, definitions of the variables, as well as the categories presented, were evaluated according to the way they were reported by the authors, including: sex (male or female), age (age group or average age), insurance (private, Medicare, Medicaid, government program, or uninsured), facility type (academic, community, or integrated network cancer program), hospital service volume (low, middle, or high), hospital level (local/regional and medical center), hospital ownership (public or private), location of initial diagnosis (diagnosis and treatment at different facilities), urbanization level of the hospital location (ranging from 1 [highly developed urban cities] to 7 [remote districts]), distance from treatment center (in miles), place of patient residence (regional/remote, metropolitan or rural, intermediate, and urban), education level (obtained by considering the percentage of individuals with a high school degree based on the patients’ residential zip code), race/skin color (white, non-white/black/Hispanic/other/unknown), and marital status (married, single, divorced, or widowed).

### Synthesis methods

The general characteristics of the articles were reported: author, year of publication, country, sample size, sex and age of the sample, definition of outcome, socioeconomic variables, and conclusion.

The individual estimates of studies that reported outcomes with odds ratios (OR) and 95% confidence intervals (95%CI) were analyzed using the STATA software, version 18 (https://www.stata.com). Final estimates were placed in a set of forest plots to visually represent the direction and magnitude of the effect ^27^. The “unknown” category was not included in the forest plot, as these data cannot be interpreted. Associations that were reported as regression coefficients or p values were presented only in the descriptive table containing all socioeconomic variables reported by all studies included in this systematic review.

The *Grading of Recommendations Assessment, Development, and Evaluation* (GRADE) approach was used to classify the quality of evidence for the outcome related to the delayed diagnosis of oral cancer, according to the following criteria: risk of bias, inconsistency, indirect evidence, inaccuracy, or publication bias. According to GRADE criteria, a level of evidence was lowered for each severe concern and observational studies started with a low classification ^28^.

### Registration and protocol

The review protocol was registered a priori in the International Prospective Register of Systematic Reviews (PROSPERO) database (registration n. #CRD42022361948).

## Results

### Article selection

**Figure 1:**
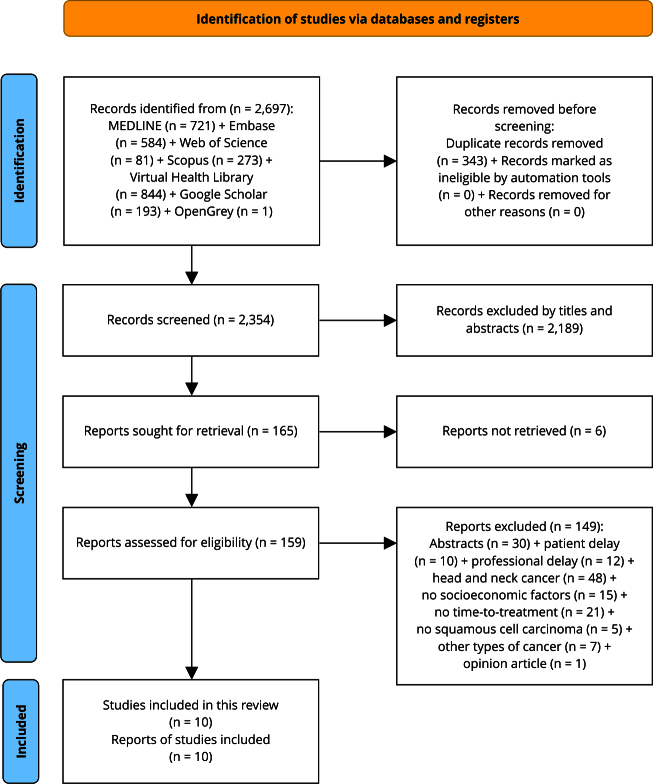
Flowchart of the screening process.

Figure 1 shows the screening process for selecting articles based on titles, abstracts, and full texts. In total, 10 retrospective cohort studies were included in this systematic review ^11^ ^,^ ^12^ ^,^ ^13^ ^,^ ^23^ ^,^ ^24^ ^,^ ^25^ ^,^ ^26^ ^,^ ^29^ ^,^ ^30^ ^,^ ^31^. Box S2 (Supplementary Material; https://cadernos.ensp.fiocruz.br/static//arquivo/suppl-e00121324_8794.pdf) presents excluded articles and the reasons for exclusion.

### Study characteristics

Box 1 shows the characteristics of the studies. All studies were published in English. Only one article was published in 2007 ^29^, whereas the others were published from 2015 to 2021. In total, five studies were conducted in the United States ^12^ ^,^ ^13^ ^,^ ^23^ ^,^ ^24^ ^,^ ^25^, two in Taiwan ^11^ ^,^ ^26^, one in Australia ^30^, one in Canada ^31^, and one in the United Kingdom ^29^.

**Box 1:** Studies characteristics and data extraction.

STUDY (YEAR) COUNTRY DATABASE	SAMPLE SIZE	SEX	AGE	CANCER SITE/TREATMENT	TIME TO TREATMENT	DEFINITION OF TREATMENT DELAY	SOCIOECONOMIC FACTORS EVALUATED	CONCLUSION
Sharma et al. ^23^ (2016); United States; NCDB	6,606	Male: 5,430 (82.2%); female: 1,176 (17.8%)	< 65 years: 5,053 (76.5%); ≥ 65 years: 1,553 (23.5%)	Oropharynx Definitive chemotherapy	Median: 32 days	Group 1: < 30 days (n = 3,020); Group 2: ≥ 30 days (n = 3,586)	Sex, age, race/skin color, insurance status, treatment facility, facility case-volume, patient distance from treatment center	Non-Hispanic black or Hispanic ethnicity, with Medicare, Medicaid coverage or uninsured status, receiving treatment at academic centers or high case-volume facilities were more likely to experience treatment delay
Morse et al. ^25^ (2018); United States; NCDB	4,089	Male: 3,387 (83%); female: 702 (17%)	≤ 60 years: 2,518 (62%); > 60 years: 1,571 (38%)	Oropharynx Radiotherapy or concurrent chemotherapy	Median: 35 days	Group 1: no delay (first and second quartiles; n = 2,012); Group 2: delay (fourth quartile; n = 1,032)	Sex, age, race/skin color, primary payor (insurance), facility type, facility volume, care transitions	Non-white skin color, with Medicaid, Medicare, or no insurance, treatment at an academic/research program, and no care transition between diagnosis and treatment were associated with treatment delay
Su et al. ^26^ (2021); Taiwan; Screening Program of the Ministry of Health and Welfare of Taiwan	5,743	Male: 5,573 (97%); female: 170 (3%)	< 30years: 17 (0.3%); 30-59 years: 4,101 (71.4%); ≥ 60 years: 1,625 (28.3%)	Oral and oropharynx NR	Median: 20 days (IQR: 12‐30)	Group 1: < 3 weeks (n = 3,267); Group 2: 3-6 weeks (n = 1,700); Group 3: 6 weeks or more (n = 776)	Sex, age, hospital level for cancer treatment	Female patients, aged 60 years or older and treated at a local/regional hospital experienced a longer delay in starting treatment
Tsai et al. ^11^ (2017); Taiwan; NHIRD/TCRD	21,263	Male: 19,505 (91.7%); female: 1,758 (8.3%)	≤ 44 years: 5,001 (23.5%); 45-54 years: 7,376 (34.7%); 55-64 years: 4,988 (23.5%); ≥ 65 years: 3,898 (18.3%)	Oral cavity Surgery, chemotherapy or radiotherapy	Mean: 24.3 days (±76.4)	Group 1: ≤ 30 days (n =18,193); Group 2: 31-120 days (n = 2,498); Group 3: > 120 days (n = 572)	Sex, age, urbanization level, premium-based monthly salary (income), hospital level, hospital ownership, hospital services volume	Female patients, ≥ 65 years, treated in regional hospitals, public hospitals and low services-volume hospitals tended to be treated later
Fujiwara et al. ^12^ (2017); United States; NCDB	4,868	Male: 2,915 (59.9%); female: 1,953 (40.1%)	< 60 years: 2,114 (43.4%); ≥ 60 years: 2,754 (56.6%)	Oral cavity Surgery + radiotherapy + chemotherapy	Median: 30 days (range: 1-731; SD = 29.3)	Delayed treatment (fourth quartile of each treatment interval) and compared the first and second quartiles	Sex, age, race/skin color, insurance, facility type, location of diagnosis	Patients above 60 years old, uninsured, insured, or using Medicaid, treatment at an academic/research program and diagnosis at an outside facility were significantly associated with prolonged diagnosis-to-surgery intervals
Morse et al. ^24^ (2018); United States; NCDB	3,708	Male: 3,022 (81%); female: 686 (19%)	≤ 60 years: 2,514 (68%); > 60 years: 1,194 (32%)	Oropharynx Primary surgery, with or without radiotherapy and chemotherapy	Median: 27 days	Delayed (fourth quartile) versus not delayed (first and second quartiles) for each interval	Sex, age, race/skin color, primary payor (insurance), facility type, facility volume, care transition	Medicaid insurance and a care transition were associated with higher chances of treatment delay
Venchiarutti et al. ^30^ (2020); Australia; Sydney Head and Neck Cancer Institute	224	Male: 162 (72.32%); female: 62 (27.67%)	Mean 60.9 years (SD = 12.1)	Oral and oropharynx NR	Overall mean: 39.5 (SD = 46.79)	Continuous variable (number of days)	Patients’ residential geocoding addresses dichotomized into metropolitan or regional/remote	Median time from diagnosis to treatment was longer for regional/remote patients’ oropharyngeal cancer. There was no difference in the median time interval for the treatment of patients with oral cavity cancer from regional/remote and metropolitan areas
Zhang et al. ^31^ (2015); Canada; ACR	554	Male: 311 (56.13%); female: 243 (43.86%)	Mean 61.3 years	Oral cancer NR	Overall mean: 51.25	Continuous variable (number of days)	Geographical groups: rural, intermediate, and urban	There was no statistically significant difference of mean time to treatment among the rural, intermediate, and urban groups. However, the urban group had the lowest (49.3) days to initiation of treatment
Rogers et al. ^29^ (2007); United Kingdom; Regional Maxillofacial Unit in Liverpool	559	Male: 343 (61%); female: 216 (39%)	Mean 61 years (SD = 13)	Oral and oropharynx NR	Median: 21 days (IQR: 12-30)	Continuous variable (median days)	Sex, age, marital status, IMD 2000	There was no difference in treatment delay time for socioeconomic variables
Goel et al. ^13^ (2019); United States; NCDB	3,550	Male: 2,937 (82.7%); female: 613 (17.3%)	≤ 50 years: 790 (22.3%); 51-60 years: 1,522 (42.9%); 61-70 years: 964 (27.2%); ≥ 71 years: 274 (7.7%)	Oropharynx Surgery resection and adjuvant radiotherapy with or without chemotherapy	Median: 26 days (IQR: 14-39)	Continuous variable (number of days)	Sex, age, race/skin color, education (percent of people with no high school degree), income (by patient’s zip code of residence), insurance, facility type	Residence in an area with low education levels, Medicaid or uninsured status and treatment at an academic facility experienced longer mean treatment delay

ACR: Alberta Cancer Registry; IMD 2000: index of multiple deprivation 2000; IQR: interquartile range; NCDB: National Cancer Database; NHIRD: National Health Insurance Research Database; NR: not reported; SD: standard deviation; TCRD: Taiwan Cancer Registry Database.

A total of six studies included samples from large national health system databases ^11^ ^,^ ^12^ ^,^ ^13^ ^,^ ^23^ ^,^ ^24^ ^,^ ^25^; one study from a national screening program ^26^; and three studies from hospital records ^29^ ^,^ ^30^ ^,^ ^31^. Sample size ranged from 224 to 21,263 participants. Male participants predominated in all studies (56.13% to 97%). Mean age ranged from 53.5 to 62.2 years in seven studies ^11^ ^,^ ^12^ ^,^ ^13^ ^,^ ^23^ ^,^ ^29^ ^,^ ^30^ ^,^ ^31^, being ≤ 60 years in the other studies ^24^ ^,^ ^25^ ^,^ ^26^. Tumor site was the oral cavity ^11^ ^,^ ^12^ ^,^ ^31^, only the oropharynx ^13^ ^,^ ^23^ ^,^ ^24^ ^,^ ^25^, or both ^26^ ^,^ ^29^ ^,^ ^30^. Initial treatment was definitive chemotherapy ^23^, radiotherapy or chemotherapy ^25^, and surgery combined or not with radiotherapy or chemotherapy ^11^ ^,^ ^12^ ^,^ ^13^ ^,^ ^24^. Type of initial therapy was not reported in four articles ^26^ ^,^ ^29^ ^,^ ^30^ ^,^ ^31^.

Median time from diagnosis to treatment ranging from 20 to 35 days was reported in seven studies ^12^ ^,^ ^13^ ^,^ ^23^ ^,^ ^24^ ^,^ ^25^ ^,^ ^26^ ^,^ ^29^. The remaining articles described a mean time ranging from 24.3 to 51.25 days ^11^ ^,^ ^30^ ^,^ ^31^. Treatment delay was heterogeneous among studies. Treatment delay was categorized by four studies as follows: Group 1: < 30 days and Group 2: ≥ 30 days ^23^; Group 1: no delay (first and second quartiles) and Group 2: delay (fourth quartile) ^25^; Group 1: 0 to 3 weeks, Group 2: 4 to 6 weeks, and Group 3: 7 or more weeks ^26^; Group 1: ≤ 30 days, Group 2: 31 to 120 days, and Group 3: > 120 days ^11^. The other studies reported treatment delay as continuous data, using mean or median number of days, quartiles, or specific number of days ^12^ ^,^ ^13^ ^,^ ^24^ ^,^ ^26^ ^,^ ^29^ ^,^ ^30^ ^,^ ^31^. Among the 10 studies, only two ^29^ ^,^ ^31^ found no association between any socioeconomic factors and treatment delay for OOSCC.

### Narrative synthesis

Box S3 (Supplementary Material; https://cadernos.ensp.fiocruz.br/static//arquivo/suppl-e00121324_8794.pdf) details the association between treatment delay and socioeconomic factors.

### Insurance

**Figure 2:**
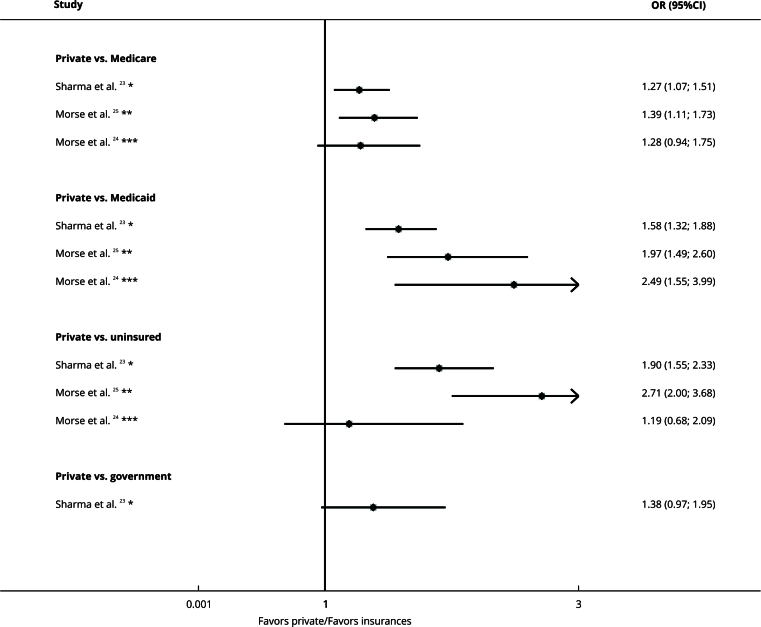
Forest plot comparing types of insurance and treatment delay organized by subgroups. Right denotes exposure, whereas left denotes the comparison.

In total, five studies conducted in the United States reported associations between treatment delay and type of insurance. Treatment delay was more likely among patients covered by Medicare (OR = 1.27, 95%CI: 1.07; 1.51) ^23^, (OR = 1.39, 95%CI: 1.11; 1.73) ^25^, or Medicaid (OR = 1.58, 95%CI: 1.32; 1.88) ^23^, (OR = 1.97, 95%CI: 1.49; 2.60) ^25^, (OR = 2.49, 95%CI: 1.55; 3.99) ^24^, and uninsured patients (OR = 1.90, 95%CI: 1.55; 2.33) ^23^, (OR = 2.71, 95%CI: 2.00; 3.68) ^25^, compared to those with private insurance ^13^ ^,^ ^23^ ^,^ ^24^ ^,^ ^25^ (Figure 2).

95%CI: 95% confidence interval; OR: odds ratio. * Delay cut-off: ≥ 30 days; ** Delay cut-off: ≥ 50 days; *** Delay cut-off: ≥ 38 days.

Goel et al. ^13^ found that Medicaid had more mean days of delay than private insurance (coefficient: 3.12, 95%CI: 0.10; 6.14) (Box 2).

The type of insurance reported as “unknown” ^12^ and associations reported as regression coefficients ^13^ are not represented in the forest plot.

The certainty of evidence was very low due to the risk of bias, inconsistency, and indirectness (Box 2).

**Box 2:** Associations between socioeconomic variables and treatment delay along with the certainty of evidence. Imported from GRADEpro Guideline Development Tool (GDT; https://gdt.gradepro.org/app/#projects).

OUTCOMES	IMPACT	TYPE OF STUDY	CERTAINTY OF EVIDENCE (GRADE)
Treatment delay Insurance (private versus Medicare)	Medicare showed more treatment delays than private insurance (Sharma et al. ^23^ - cut-off 30 days, OR = 1.27, 95%CI: 1.07; 1.51; Morse et al. ^25^ - cut-off: 50 days, OR = 1.39, 95%CI: 1.11; 1.73). However, Morse et al. ^24^ (cut-off: 38 days) found that private insurance shows slightly less delay or is similar to Medicare (OR = 1.28, 95%CI: 0.94; 1.75). Goel et al. ^13^ (cut-off 30 days) found no significant differences in mean days of delay for private and Medicare (coefficient: 1.14, -0.98; 3.00)	4 non-randomized	⨁◯◯◯ Very low * ** ***
Treatment delay Insurance (private versus Medicaid)	Medicaid showed more treatment delays than private insurance (Sharma et al. ^23^ - cut-off 30 days, OR = 1.58, 95%CI: 1.32; 1.88; Morse et al. ^25^ - cut-off: 50 days, OR = 1.97, 95%CI: 1.49; 2.60; Morse et al. ^24^ - cut-off: 38 days, OR = 2.49, 95%CI: 1.55; 3.99). Also, Goel et al. ^13^ (cut-off 30 days) showed that Medicaid had more mean days of delay than private insurance (coefficient: 3.12, 0.10; 6.14)	4 non-randomized	⨁◯◯◯ Very low * ** ***
Treatment delay Insurance (private versus government program)	Government programs showed more treatment delays than private (Sharma et al. ^23^ - cut-off 30 days, OR = 1.38, 95%CI: 0.97; 1.95)	1 non-randomized	⨁◯◯◯ Very low ***
Treatment delay Insurance (private versus uninsured)	Uninsured showed more treatment delays than private insurance (Sharma et al. ^23^ - cut-off 30 days, OR = 1.90, 95%CI: 1.55; 2.33; Morse et al. ^25^ - cut-off: 50 days, OR = 2.71, 95%CI: 2.00; 3.68; Goel et al. ^13^ - cut-off 30 days, coefficient: 4.95, 0.71; 9.20). However, uninsured showed a similar delay compared to private insurance (Morse et al. ^24^ - cut-off: 38 days, OR = 1.19, 95%CI: 0.68; 2.09)	4 non-randomized	⨁◯◯◯ Very low * ** ***
Treatment delay Facility type (community versus academic)	Academic medical centers showed more treatment delays than community centers (Sharma et al. ^23^ - cut-off 30 days, OR = 1.26, 95%CI: 1.13; 1.42; Morse et al. ^25^ - cut-off: 50 days, OR = 1.52, 95%CI: 1.03; 2.26; Fujiwara et al. ^12^ - cut-off: 45 days, OR = 2.17, 95%CI: 1.49; 3.15), being also found to show more or equivalent delay (Morse et al. ^24^ - cut-off: 38 days, OR = 1.79, 95%CI: 0.92; 3.51). Goel et al. ^13^ (cut-off 30 days) found that community centers show less mean days of delay than academic (coefficient: -5.28, -8.57; -1.99)	5 non-randomized	⨁◯◯◯ Very low * ** ***
Treatment delay Facility type (community versus comprehensive)	Comprehensive community cancer programs showed similar delays when compared to community programs (Morse et al. ^25^ - cut-off: 50 days, OR = 0.98, 95%CI: 0.68; 1.42; Fujiwara et al. ^12^ - cut-off: 45 days, OR = 1.16, 95%CI: 0.79; 1.70; Morse et al. ^24^ - cut-off: 38 days, OR = 1.24, 95%CI: 0.65; 2.35).	4 non-randomized	⨁◯◯◯ Very low * ** ***
Treatment delay Facility type (community versus integrated network cancer program)	Integrated network cancer program showed greater or similar delays when compared to community programs (Morse et al. ^25^ - cut-off: 50 days, OR = 1.33, 95%CI: 0.85; 2.08; Morse et al. ^24^ - cut-off: 38 days, OR = 1.38, 95%CI: 0.65; 2.93).	2 non-randomized	⨁◯◯◯ Very low * ** ***
Treatment delay Facility type (academic versus comprehensive)	Academic facilities showed more mean days of delay than comprehensive programs (Goel et al. ^13^ - cut-off:30 days, coefficient: -4.95, 95%CI: -6.65; -3.25)	1 non-randomized	⨁◯◯◯ Very low * ** ***
Treatment delay Hospital services volume (low versus high)	High-volume hospital services were found to present more delay than low-volume services in a study (Sharma et al. ^23^ - cut-off 30 days, OR = 1.38, 95%CI: 1.21; 1.58). However, high-volume services were found to show similar results compared to low-volume services in another study (Morse et al. ^25^ - cut-off: 50 days, OR: 0.93, 95%CI: 0.67; 1.29; Morse et al. ^24^ - cut-off: 38 days, OR = 1.09, 95%CI: 0.65; 1.83). Tsai et al. ^11^ showed that low-volume services was associated with a longer time to treatment (p < 0.001)	4 non-randomized	⨁◯◯◯ Very low * ** ^#^
Treatment delay Hospital services volume (low versus moderate)	Moderate-volume hospitals showed similar treatment delay when compared to low-services (Morse et al. ^25^ - cut-off: 50 days, OR = 0.81, 95%CI: 0.62; 1.07; Morse et al. ^24^ - cut-off: 38 days, OR = 1.09, 95%CI: 0.68; 1.76)	2 non-randomized	⨁◯◯◯ Very low * ** ***
Treatment delay Hospital level (medical centers versus local/regional)	Patients treated at local/regional hospitals showed longer delays compared to those treated at hospital centers (Su et al. ^26^ - cut-off: > 6 weeks, p < 0.001; Tsai et al. ^11^ - cut-off: 30 days, p = 0.02)	2 non-randomized	⨁◯◯◯ Very low * ** ***
Treatment delay Hospital ownership (public versus private)	Patients treated at a public hospital showed more treatment delays compared to those treated at a private hospital (Tsai et al. ^11^ - cut-off: 30 days, p < 0.001)	1 non-randomized	⨁◯◯◯ Very low * ***
Treatment delay Care transition (care transition versus no care transition)	Studies reported varying results. Morse et al. ^25^ (cut-off: 50 days) found that patients who did not need a care transition were more likely to experience treatment delay (OR = 1.73, 95%CI: 1.46; 2.04) and Morse et al. ^24^ (cut-off: 38 days) found that those who needed a care transition were more than 2-fold more likely to experience treatment delay (OR = 2.70, 95%CI: 2.1; 3.49)	2 non-randomized	⨁◯◯◯ Very low * ** ***
Treatment delay Care transition (at reporting facility versus outside facility)	Location of diagnosis at an outside facility were more than two-fold more likely to experience treatment delay (Fujiwara et al. ^12^ - cut-off: 45 days, OR: 2.17, 95%CI: 1.49; 3.15)	1 non-randomized	⨁◯◯◯ Very low * ***
Treatment delay Urbanization level hospital (levels 1, 2&3, 4&5, 6&7)	The level of urbanization where the hospital was located was not associated with treatment delay (Tsai et al. ^11^ - cut-off: 30 days, p = 0.951)	1 non-randomized	⨁◯◯◯ Very low *** ^##^
Treatment delay Distance from treatment center (< 25 miles versus 25-100 miles)	Sharma et al. ^23^ (cut-off: 30 days) showed that distance < 25 miles from the patient’s place of residence to the treatment center showed a similar delay compared to 25-100 miles (OR = 1.00, 95%CI: 0.88; 1.14)	1 non-randomized	⨁◯◯◯ Very low * ***
Treatment delay Distance from treatment center (< 25 miles versus > 100 miles)	Sharma et al. ^23^ (cut-off: 30 days) showed that distance < 25 miles from the patient’s place of residence to the treatment center showed a similar delay compared to > 100 miles (OR = 1.08, 95%CI: 0.83; 1.41)	1 non-randomized	⨁◯◯◯ Very low ***
Treatment delay Place of residence (metropolitan versus regional/remote)	Venchiarutti et al. ^30^ (cut-off: 4 months) showed that patients living in a regional/remote area experienced a higher median treatment delay than those in a metropolitan area for oropharyngeal cancer (p = 0.03), but not for oral cavity cancer (p = 0.19)	1 non-randomized	⨁◯◯◯ Very low * ***
Treatment delay Place of residence (rural, intermediate, urban)	Zhang et al. ^31^ found no significant difference in mean days of treatment delay among patients living in rural, intermediate, and urban areas (p = 0.791)	1 non-randomized	⨁◯◯◯ Very low * ***
Treatment delay Income (low-income, ≤ 17,280 TWD, 17,281 to 22,800 TWD, ≥ 22,801 TWD)	Tsai et al. ^11^ (cut-off: 30 days) found no significant difference in treatment delay between low- and high-income patients, categorized as ≤ 17,280 TWD, 17,281 to 22,800 TWD, and ≥ 22,801 TWD (p = 0.161)	1 non-randomized	⨁◯◯◯ Very low * ***
Treatment delay Income (< 38,000 USD versus 38,000 to 47,999 USD)	Median household income of < 38,000 USD experienced a delay similar to 38,000-47,999 USD (Goel et al. ^13^ - cut-off: 30 days, coefficient: -0.11, 95%CI: -2.87; 2.64)	1 non-randomized	⨁◯◯◯ Very low * ***
Treatment delay Income (< 38,000 USD versus 48,000 to 62,999 USD)	Median household income of < 38,000 USD experienced a delay similar to 48,000-62,999 USD (Goel et al. ^13^ - cut-off: 30 days, coefficient: 0.78, 95%CI: -1.99; 3.56)	1 non-randomized	⨁◯◯◯ Very low * ***
Treatment delay Income (< 38,000 USD versus ≥ 63,000 USD)	Median household income of < 38,000 USD experienced a mean delay similar to ≥ 63,000 USD (Goel et al. ^13^ - cut-off: 30 days, coefficient: 0.55, 95%CI: -2.45; 3.55)	1 non-randomized	⨁◯◯◯ Very low * ***
Treatment delay Income (IMD 2000 deprivation tertile groups: median rank 149, median rank 1,308, median rank 4,642)	The IMD 2000. There are IMD national ranks attached to each ward of residence. The most deprived rating for each domain was 1 and the least deprived was 8,414. Rogers et al. ^29^ found no difference in median days of delay across IMD 2000 tertile groups: median rank 149, median rank 1,308, and median rank 4,642 (p = 0.50)	1 non-randomized	⨁◯◯◯ Very low * ***
Treatment delay Education (≥ 21% versus 13% to 20.9%)	Patients living in areas where ≥ 21% of residents had a high school degree experienced a similar mean treatment delay compared to those where 13% to 20.9% of residents had a high school degree (Goel et al. ^13^ - cut-off: 30 days, coefficient: -2.01, 95%CI: -4.77; 0.75)	1 non-randomized	⨁◯◯◯ Very low * ***
Treatment delay Education (≥ 21% versus 7% to 12.9%)	Patients living in areas where ≥ 21% of residents had a high school degree experienced less mean delay compared to those where 7% to 12.9% of residents had a high school degree (Goel et al. ^13^ - cut-off: 30 days, coefficient: -3.11, 95%CI: -6.04; 0.21)	1 non-randomized	⨁◯◯◯ Very low * ***
Treatment delay Education (≥ 21% versus < 7%)	Patients living in areas where ≥ 21% of residents had a high school degree experienced less mean delay compared to those where < 7% had a high school degree (Goel et al. ^13^ - cut-off: 30 days, coefficient: -6.42, 95%CI: -9.61; -3.22)	1 non-randomized	⨁◯◯◯ Very low * ***
Treatment delay Race/Skin color (non-Hispanic white versus non-Hispanic black)	Non-Hispanic blacks experienced more treatment delays compared to non-Hispanic whites (Sharma et al. ^23^ - cut-off: 30 days, OR = 1.24, 95%CI: 1.04; 1.46)	1 non-randomized	⨁◯◯◯ Very low ***
Treatment delay Race/Skin color (non-Hispanic white versus Hispanic)	Hispanic had more delay than non-Hispanic white (Sharma et al. ^23^ - cut-off: 30 days, OR = 1.74, 95%CI: 1.29; 2.34)	1 non-randomized	⨁◯◯◯ Very low ***
Treatment delay Race/Skin color (non-white versus white)	White had less delay than non-white (Morse et al. ^25^ - cut-off: 50 days, OR = 0.69, 95%CI: 0.54; 0.88). However, white had similar delay to non-white for Morse et al. ^24^ (cut-off: 38 days, OR = 0.88, 95%CI: 0.59; 1.31) and for Fujiwara et al. ^12^ (cut-off: 45 days, OR = 0.96, 95%CI: 0.77; 1.21)	3 non-randomized	⨁◯◯◯ Very low * ** ***
Treatment delay Race/Skin color (white versus black)	No significant difference was found in the mean days of delay between whites and blacks (Goel et al. ^13^ - cut-off 30 days, coefficient: 1.44; 95%CI: -2.27; 5.16)	1 non-randomized	⨁◯◯◯ Very low * ***
Treatment delay Marital status (married, single, divorced, and widowed)	No significant difference was found in delay between types of marital status (married, single, divorced, and widowed) (Rogers et al. ^29^ - p = 0.62)	1 non-randomized	⨁◯◯◯ Very low * ***
Treatment delay Sex (male versus female)	Females experienced slightly less or similar treatment delay compared to males (Sharma et al. ^23^ - cut-off 30 days, OR = 0.99, 95%CI: 0.84; 1.18; Morse et al. ^25^ - cut-off: 50 days, OR = 1.22, 95%CI: 0.98; 1.51; and Morse et al. ^24^ - cut-off: 38 days, OR = 1.05, 95%CI: 0.79; 1.41). Moreover, two studies found a tendency for delayed treatment among females (Su et al. ^26^ - cut-off: > 6 weeks, p = 0.008) and Tsai et al. ^11^ - cut-off: 30 days, p = 0.010)	5 non-randomized	⨁◯◯◯ Very low * **
Treatment delay Age (≤ 60 years versus > 60 years)	Patients aged ≤ 60 years experienced slightly less delay or similar delay compared to those aged > 60 years (Morse et al. ^25^ - cut-off: 50 days, OR = 0.96, 95%CI: 0.79; 1.17; and Morse et al. ^24^ - cut-off: 38 days, OR = 0.96, 95%CI: 0.73; 1.27)	2 non-randomized	⨁◯◯◯ Very low * ** ***
Treatment delay Age (< 60 years versus ≥ 60 years)	Patients aged ≥ 60 years experienced more delay compared to those aged < 60 years (Fujiwara et al. ^12^ - cut-off: 45 days, OR = 1.22, 95%CI: 1.01; 1.47). Moreover, Su et al. ^26^ (cut-off: > 6 weeks) showed that patients ≥ 60 years experienced a longer delay (p = 0.02)	2 non-randomized	⨁◯◯◯ Very low * **

95%CI: 95% confidence interval; IMD 2000: index of multiple deprivation 2000; OR: odds ratio.

* Severe risk of bias issues. Some studies did not adjust for confounding factors;

** Severe issues due to inconsistency: variations in studies occurred due to varying cut-off point for treatment delay;

*** Severe issues of indirectness: the studies represent a single country;

^#^ Very severe issues due to inconsistency: varying cut-off points (delay) and methodologies for categorizing the exposure. The authors classified service volume differently: Sharma et al. ^23^ divided study patients per facility by quartiles (upper quartile were considered high volume), Morse et al. ^25^ considered the number of cases treated per year (9: high-volume; 6 to 9: medium-volume; and < 6: low-volume), Tsai et al. ^11^ considered the number of services provided (low: lowest quartile; moderate: second and third quartile; and high: highest quartile);

^##^ Very severe risk of bias issues: there was no adjustment for confounding factors. There was a lack of clarity in categorizing the variable into urbanization levels from 1 to 7.

### Facility type

**Figure 3:**
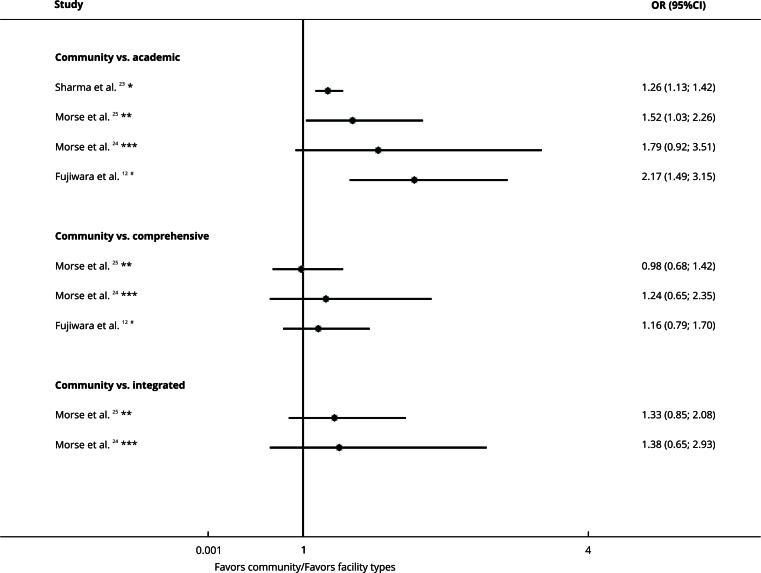
Forest plot comparing facility type and treatment delay organized by subgroups.

A total of five articles conducted in the United States reported on facility type. From these, three studies found that academic/research programs had a greater chance of treatment delay (OR = 1.26, 95%CI: 1.13; 1.42) ^23^, (OR = 1.52, 95%CI: 1.03; 2.26) ^25^, (OR = 2.17, 95%CI: 1.49; 3.15) ^12^ compared to community facilities ^23^ and comprehensive community cancer programs ^12^ ^,^ ^25^. Community, comprehensive community ^12^ ^,^ ^24^ ^,^ ^25^, and integrated network cancer ^24^ ^,^ ^25^ facilities showed similar delays (Figure 3).

95%CI: 95% confidence interval; OR: odds ratio. Note: insurance type on the right denotes exposure, whereas left denotes the comparison. * Delay cut-off: ≥ 30 days; ** Delay cut-off: ≥ 50 days; *** Delay cut-off: ≥ 38 days; ^#^ Delay cut-off: ≥ 45 days.

Goel et al. ^13^ found that comprehensive community facilities (coefficient: -4.95, 95%CI: -6.65; -3.25) and community facilities (coefficient: -5.28, 95%CI: -8.57; -1.99) had shorter treatment delays compared to academic programs (Box 2).

The certainty of evidence was very low due to the risk of bias, inconsistency, and indirectness (Box 2).

### Hospital services volume

**Figure 4:**
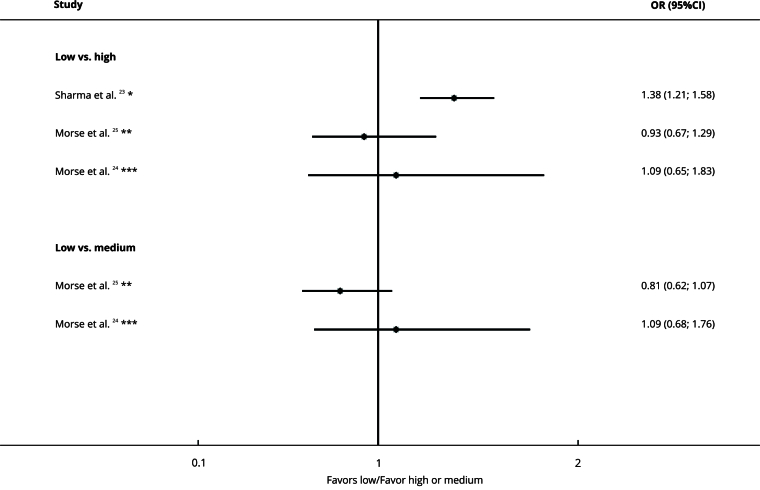
Forest plot comparing hospital services volume and treatment delay organized by subgroups.

Sharma et al. ^23^ found that high-volume hospitals were more likely to have treatment delays than low-volume hospitals (OR = 1.38, 95%CI: 1.21; 1.58). However, other studies reported no significant difference between high- and low-volume services (OR = 0.93, 95%CI: 0.67; 1.29 ^25^ and OR = 1.09, 95%CI: 0.65; 1.83 ^24^) (Figure 4).

95%CI: 95% confidence interval; OR: odds ratio. Note: insurance type on the right denotes exposure, whereas left denotes the comparison. * Delay cut-off: ≥ 30 days; ** Delay cut-off: ≥ 50 days; *** Delay cut-off: ≥ 38 days.

Tsai et al. ^11^ concluded that low-volume hospital service was associated with a longer time prior to the onset of treatment (p < 0.001) (Box 2).

The certainty of evidence was very low due to the risk of bias, inconsistency, and indirectness (Box 2).

### Hospital level

In total, two studies from Taiwan concluded that patients treated at regional hospitals were more likely to have longer delays than those treated at hospital centers (p < 0.001 ^11^ and p = 0.02 ^26^) (Box 2).

The certainty of evidence was very low due to the risk of bias, inconsistency, and indirectness (Box 2).

### Type of hospital facility

A study from Taiwan ^11^ showed that patients treated at a public hospital were more likely to have treatment delay compared to those treated at a private hospital (p < 0.001) (Box 2).

The certainty of evidence was very low due to the risk of bias and indirectness (Box 2).

### Care transition/location of initial diagnosis

**Figure 5:**
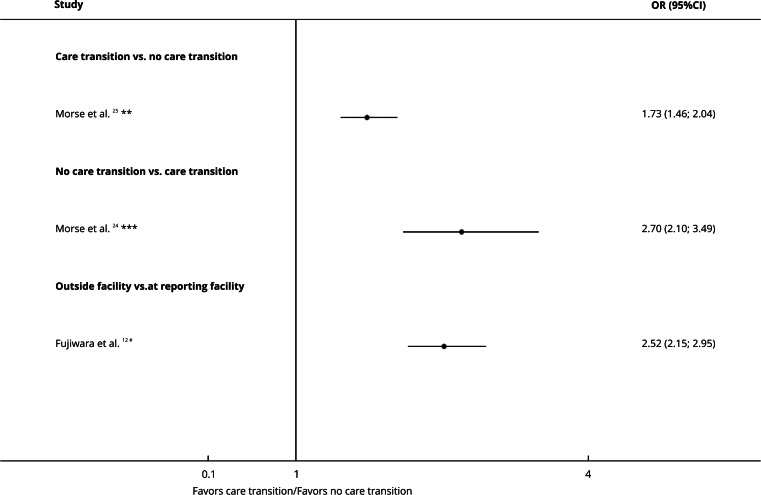
Forest plot comparing need for care transition and treatment delay organized by subgroups.

In total, three studies conducted in the United States found an association between treatment delay and the need for referral for treatment at a different facility than that of diagnosis ^12^ ^,^ ^24^ ^,^ ^25^. However, the studies reported different results. From these, one study found that patients who did not need a care transition were more likely to have treatment delay (OR = 1.73, 95%CI: 1.46; 2.04) ^25^, whereas the other studies concluded that those who needed an outside facility were more than 2-fold more likely to have treatment delay (OR = 2.52, 95%CI: 2.15; 2.95 ^12^ and OR = 2.70, 95%CI: 2.10; 3.49 ^24^) (Figure 5).

95%CI: 95% confidence interval; OR: odds ratio. Note: insurance type on the right denotes exposure, whereas left denotes the comparison. * Delay cut-off: ≥ 50 days; ** Delay cut-off: ≥ 38 days; *** Delay cut-off: ≥ 45 days.

The certainty of evidence was very low due to the risk of bias, inconsistency, and indirectness (Box 2).

### Urbanization level hospital

The level of urbanization seems to show no significant impact on treatment delays. A study conducted in the Taiwan ^11^ assessed the level of urbanization where the hospital was located and found no association with treatment delay (p = 0.951).

The certainty of evidence was very low due to the risk of bias and indirectness (Box 2).

### Distance from treatment center

The distance (in miles) from the patient’s place of residence to the treatment center was not associated with treatment delay in the United States (p > 0.05) ^23^.

The certainty of evidence was very low due to the risk of bias and indirectness (Box 2).

### Place of residence

In Australia, patients living in a regional/remote area had a higher median treatment delay than those in a metropolitan area. However, the difference was only for oropharyngeal cancer (p = 0.03) ^30^ and not for oral cavity cancer (p = 0.19) ^30^ (Box 2).

A study conducted in Canada ^31^ found no difference in mean days of treatment delay among patients who resided in rural, intermediate, and urban areas (p = 0.791) (Box 2).

The certainty of evidence was very low due to the risk of bias, inconsistency, and indirectness (Box 2).

### Income

In total, three studies investigated patient income, and none found an association with treatment delay (p = 0.161 ^11^, p > 0.05 ^13^, and p = 0.50 ^29^). Furthermore, these studies categorized this variable differently according to the economic reality of each country (Taiwan, the United States, and the United Kingdom, respectively).

Thus, the certainty of evidence was very low due to the risk of bias, inconsistency, and indirectness (Box 2).

### Education

A study conducted in the United States ^13^ reported that patients living in areas where 7% to 12.9% of residents with a high school degree (coefficient: -3.12, 95%CI: -6.04; -0.21) or < 7% (coefficient: -6.42, 95%CI: -9.61; -3.22) had longer average treatment delays compared to areas where ≥ 21% of residents had a high school degree (Box 2).

The certainty of evidence was very low due to the risk of bias and indirectness (Box 2).

### Race/Skin color

**Figure 6:**
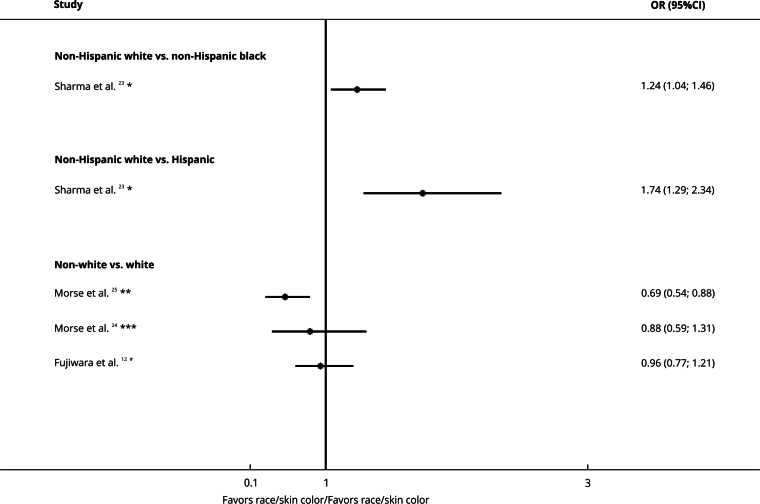
Forest plot comparing race/skin color and treatment delay organized by subgroups.

Race/skin color was investigated in five U.S. studies. From these, one study ^23^ showed that non-whites were more likely to have treatment delay (OR = 1.74, 95%CI: 1.29; 2.34 for Hispanics and OR = 1.24, 95%CI: 1.04; 1.46 for non-Hispanic blacks). Another study ^25^ reported a lower likelihood of delay among whites (OR = 0.69, 95%CI: 0.54; 0.88). However, whites and non-whites had similar delay in two studies ^12^ ^,^ ^24^ (Figure 6).

95%CI: 95% confidence interval; OR: odds ratio. Note: insurance type on the right denotes exposure, whereas left denotes the comparison. * Delay cut-off: ≥ 30 days; ** Delay cut-off: ≥ 50 days; *** Delay cut-off: ≥ 38 days; ^#^ Delay cut-off: ≥ 45 days.

The certainty of evidence was very low due to the risk of bias, inconsistency, and indirectness (Box 2).

### Marital status

No association was found between marital status and median days of treatment delay (p = 0.62) ^29^ (Box 2).

The certainty of evidence was very low due to the risk of bias and indirectness (Box 2).

### Sex and age

**Figure 7:**
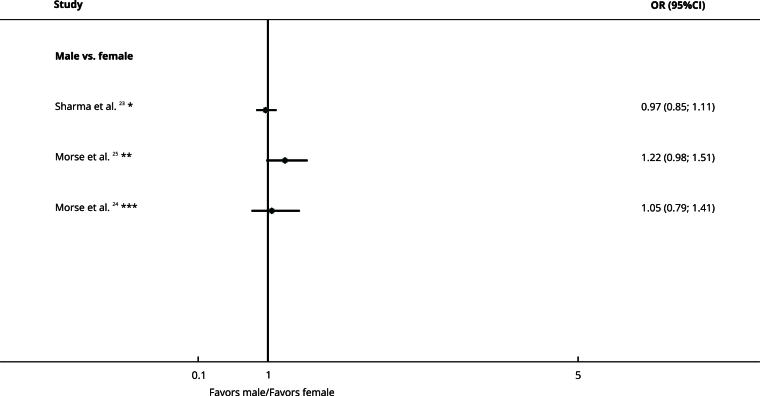
Forest plot comparing sex and treatment delay organized by subgroups.

**Figure 8:**
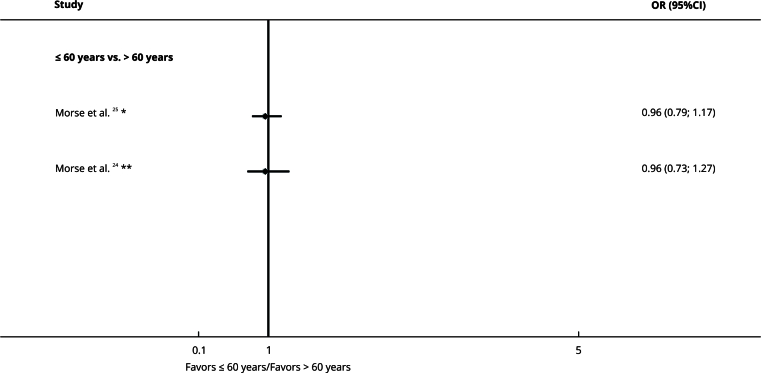
Forest plot comparing age and treatment delay organized by subgroups.

Sex (OR = 0.97, 95%CI: 0.85; 1.11 ^23^; OR = 1.22, 95%CI: 0.98; 1.51 ^25^; OR = 1.05, 95%CI: 0.79; 1.41 ^24^) (Figure 7) and age (OR = 0.96, 95%CI: 0.79; 1.17 ^25^, OR = 0.96, 95%CI: 0.73; 1.27 ^24^) (Figure 8) did not impact treatment delay.

95%CI: 95% confidence interval; OR: odds ratio. Note: insurance type on the right denotes exposure, whereas left denotes the comparison. * Delay cut-off: ≥ 30 days; ** Delay cut-off: ≥ 50 days; *** Delay cut-off: ≥ 38 days.

95%CI: 95% confidence interval; OR: odds ratio. Note: insurance type on the right denotes exposure, whereas left denotes the comparison. * Delay cut-off: ≥ 50 days; ** Delay cut-off: ≥ 38 days.

The certainty of evidence was very low due to the risk of bias, inconsistency, and indirectness (Box 2).

### Risk of bias

The most common methodological issues were related to Item 4 (identification of confounders) and Item 5 (strategies for dealing with confounding factors), both rated “no” in nine studies. Domain 10 (strategies for addressing incomplete follow-up) was rated “no” in three studies. Domains 9 (reasons for losses to follow-up described and explored) and 10 (strategies for addressing incomplete follow-up) were considered “not applicable” in six studies that defined missing or incomplete data as exclusion criteria. In total, two studies were rated as “unclear” for Domain 3 (exposure measured in a valid and reliable way), as treatment delay was defined as the fourth quartile, but the precise value of each quartile was unclear (Supplementary Material - Box S4; https://cadernos.ensp.fiocruz.br/static//arquivo/suppl-e00121324_8794.pdf).

## Discussion

Most studies found an association between some socioeconomic factors and delayed treatment of OOSCC. However, the certainty of these associations was very low.

The findings regarding the type of insurance were derived exclusively from studies conducted in the United States ^12^ ^,^ ^13^ ^,^ ^23^ ^,^ ^24^ ^,^ ^25^. Consequently, we suggest caution when interpreting these findings, as they regard the health system of a single country. The U.S. healthcare system is a hybrid of private health insurance and government programs ^32^ ^,^ ^33^. The U.S. government insurance (Medicaid, Medicare, and other programs) was more frequently associated with treatment delay compared to private insurance.

It seems reasonable to conclude that individuals who rely on government-funded healthcare or are uninsured are socioeconomically disadvantaged and unable to afford the high costs associated with OOSCC treatment. The economic burden may explain the unfeasibility of timely treatment ^32^. However, other factors must be considered. Medicare primarily serves individuals aged 65 and older who have paid taxes on their earnings, thus these patients often present with comorbidities that require treatment before commencing definitive OOSCC therapy ^33^ ^,^ ^34^. Individuals utilizing Medicaid (main insurance for low-income people), along with those who are uninsured, tend to have more advanced disease stages, potentially due to heightened exposure to health risks and limited access to preventive care ^35^ ^,^ ^36^ ^,^ ^37^.

Therefore, depending on the patient’s profile and the complexity of cases that require advanced (radiotherapy or chemotherapy) or multimodal (surgery combined with radiotherapy and/or chemotherapy) therapies, it is likely that additional tests and comorbidity adjustments will be required prior to treatment, leading to longer delays ^38^. Similarly, cases that require care transition also experience longer delays ^12^ ^,^ ^24^.

At the same time, a study from Taiwan, which has a different healthcare system than the United States ^39^, found that public hospitals were more frequently associated with delayed treatment than private hospitals. However, this study did not control for confounders of disease/treatment. According to the analyses, the group with the longest time to treatment (> 120 days) had a higher percentage of advanced stages (III and IV) and required radiotherapy and chemotherapy treatments. This suggests that the delay in treatment may not necessarily be due to the service provided by these facilities, but rather to the lack of early detection due to gaps in knowledge about the disease among the population that depends on the public health system ^40^. In other words, people with low socioeconomic status often fail to notice malignant lesions in their early stages and delay seeking healthcare ^41^, resulting in advanced stages at presentation. As advanced stages require more pre-treatment care, this impacts treatment delay.

It is crucial to note that tobacco/alcohol use may also be important confounding factors not identified by the studies in this review. In general, patients who are heavy smokers and alcohol users tend to have low socioeconomic status and are often uncooperative with treatment plans or unreliable in keeping appointments ^35^. Thus, the fact that the utilization of services for OOSCC also depends on the patient’s self-perception of the need to cooperate in order to receive timely treatment cannot be overlooked ^9^ ^,^ ^41^.

The studies that evaluated type of facilities are also from the United States ^12^ ^,^ ^13^ ^,^ ^23^ ^,^ ^24^ ^,^ ^25^. Academic medical centers tend to be located in large urban centers, attending a high number of underprivileged communities and lower socioeconomic status individuals (uninsured and government insured). They also serve as referral centers for other hospitals that lack the equipment and personnel to manage OOSCC ^42^. This suggests that the greater backlog at academic medical centers, as revealed by the studies in this review, may not be due to the facilities themselves, but rather the high demand for OOSCC treatment added to the profile of these patients, which often includes preexisting health conditions and precarious living situations ^14^ ^,^ ^42^.

The volume of hospital services showed conflicting results: a high volume of patients seen in the hospital was associated with delayed treatment ^23^. However, a low volume of hospital procedures offered was associated with delays in one study ^11^, whereas another found no association ^25^. It is possible that the heterogeneity of the authors’ definition of the variable and/or the organization of healthcare systems in different countries (United States ^23^ ^,^ ^25^ and Taiwan ^11^) may have impacted these results.

Regarding place of residence, a Canadian study ^31^ found no difference in treatment delay for oral cancer patients living in rural, intermediate, or urban areas. According to the authors, the organization of the healthcare system may allow for greater standardization of treatment among patients regardless of where they live, which may explain this finding. On the other hand, an Australian study ^30^ showed a greater delay in oropharyngeal cancer treatment for patients living in remote areas compared to those in metropolitan areas, but did not find this difference for oral cancer.

In this case, it is important to note that cancer site must be considered. It is possible that in countries with a high level of socioeconomic development, such as Canada and Australia, both of which have universal healthcare systems, place of residence does not impact delays in treatment for oral cancer, which is often treated with surgery alone ^43^. However, when more complex therapies are required (such as radiotherapy for oropharyngeal cancer), the greater need for referrals to different services for pre-treatment care creates accessibility barriers for people who live farther away, leading to longer treatment times ^44^.

Studies conducted in the United States have considered two factors related to social class, namely education and race/skin color. Patients living in areas with a higher percentage of individuals with low education ^13^ and non-white individuals ^23^ ^,^ ^25^ were more likely to have delayed treatment. Although these studies show a high risk of bias, particularly because they did not evaluate the education and income levels of individuals, ethnic and educational disparities in access to cancer care have been reported previously ^14^ ^,^ ^45^ ^,^ ^46^. People with lower levels of education and non-whites were more likely to have inadequate information and lower levels of knowledge about OOSCC, leading to late presentation and more advanced disease stages ^47^, as well as greater refusal or resistance to recommended treatments ^48^.

None of the studies that assessed income found an association with treatment delay ^11^ ^,^ ^13^ ^,^ ^29^. In studies conducted in the United States ^13^ and the United Kingdom ^29^, the assessment of this variable was limited to the geographic area and rather than the individual’s income (e.g., average household income in the patient’s zip code of residence) ^13^. Therefore, it cannot be assumed that everyone living in a deprived area is deprived, or vice versa. Similarly, it is not possible to conclude that an individual’s income does not impact time to treatment for OOSCC, even in rich country contexts. A study conducted in Taiwan assessed patients’ monthly income but did not adjust for confounders. The authors concluded that the lack of difference in treatment delay between low-income and high-income patients may be explained by the universal public healthcare system in the country. In that system, cancer patients are exempt from co-payments for treatment, and low-income patients receive subsidies to ensure their right to adequate health care ^39^.

Only two studies from Taiwan ^11^ ^,^ ^26^ found female sex to be associated with treatment delay, and three studies ^11^ ^,^ ^12^ ^,^ ^26^ found an association between older age and treatment delay. However, these results may have been confounded by the various characteristics of the disease and therapeutic protocols.

### Limitations

The overall risk of bias in the studies included in this systematic review was assessed as high. Although the JBI tool holds limitations, as it refers to the potential for bias rather than the actual measurement of bias in the studies, and does not include the assessment of other constructs such as reporting quality, random error, external validity, or ethical considerations, it is still recommended as a valid tool for assessing the risk of bias in line with the GRADE approach classification domains ^49^.

Thus, severe bias issues were found in the studies included, as nine of ten did not identify or address confounding factors ^11^ ^,^ ^12^ ^,^ ^13^ ^,^ ^24^ ^,^ ^25^ ^,^ ^26^ ^,^ ^29^ ^,^ ^30^ ^,^ ^31^, and three did not implement strategies for dealing with incomplete follow-up ^11^ ^,^ ^13^ ^,^ ^25^. The variables were approached by the authors according to the information available in secondary databases, which did not always include key determinants for assessing access to healthcare services, such as education, occupation, place of residence, and income ^9^.

All studies were conducted in countries with high economic and social development, including the United States ^12^ ^,^ ^13^ ^,^ ^23^ ^,^ ^24^ ^,^ ^25^, the United Kingdom ^29^, Taiwan ^11^ ^,^ ^26^, Australia ^30^, and Canada ^31^. However, comparisons were not feasible due to significant heterogeneity in the definition of treatment delay, leading to severe issues of inconsistency. Additionally, socioeconomic variables were defined and categorized differently across studies, often according to the organizational structure of each national healthcare system and geographic classification criteria. This diversity of definitions prevented a grouped analysis.

Similarly, the limited number of studies examining the relationship between socioeconomic factors and delayed OOSCC treatment led to severe issues of indirect evidence. This also limits the generalizability of the findings to diverse healthcare settings, particularly in countries with lower socioeconomic development indicators.

Due to the considerable heterogeneity in the categorization of treatment delay and outcomes across studies, it was not feasible to conduct a meta-analysis or subgroup analysis, such as by sex, race/skin color, age, insurance type, or other factors. Although language restrictions were not applied as exclusion criteria, only studies in English were retrieved. Moreover, the impossibility of assessing publication bias and the limitations inherent in the included studies may have impacted the results of our research.

### Strengths

Most studies on “delay” address head and neck cancer. This systematic review included OOSCC, including only studies that clearly defined the time to treatment onset following the Aarhus model ^5^.

Although the studies significantly varied in the categorization of treatment delay and outcomes, we synthesized all available results in a narrative format.

## Conclusion

An association was found between treatment delay of OOSCC and various socioeconomic factors, such as type of insurance, sex, older age, non-white skin color, low education levels, treatment in public or regional hospital, need for transition care, and living in regional/remote areas. However, the available data do not support a definitive conclusion regarding this association. High methodological discrepancies across studies ₋ particularly in defining treatment delay and categorizing socioeconomic variables ₋ along with limited number of studies, contributed to a very low certainty of evidence.

### Implications for practice and future studies

Considering that treatment delay can be used as a quality indicator to monitor service performance, healthcare providers ₋ both public (government) and private ₋ should make efforts to reduce delays in care for patients with OOSCC.

Therefore, future studies are encouraged in different countries with varying healthcare systems and socioeconomic contexts to provide a deeper understanding of the determinants of treatment delay in OOSCC. Studies should focus on OOSCC treatment delay as the primary outcome, defined as the time from definitive diagnosis to the first intervention ^5^ ^,^ ^6^. Methodological quality must be improved, with more accurate comparisons of delay and non-delay groups. It is also important to clearly define the sociodemographic and economic variables of the participants to obtain more consistent and applicable results for clinical decisions.

## References

[1] Hung LC, Kung PT, Lung CH, Tsai MH, Liu SA, Chiu LT (2020). Assessment of the risk of oral cancer incidence in a high-risk population and establishment of a predictive model for oral cancer incidence using a population-based cohort in Taiwan. Int J Environ Res Public Health.

[2] Nagao T, Warnakulasuriya S (2020). Screening for oral cancer future prospects, research and policy development for Asia. Oral Oncol.

[3] Lins LS, Bezerra NV, Freire AR, Almeida LD, Lucena EH, Cavalcanti YW (2019). Socio-demographic characteristics are related to the advanced clinical stage of oral cancer. Med Oral Patol Oral Cir Bucal.

[4] Herrera-Serna BY, Lara-Carrillo E, Toral-Rizo VH, Cristina do Amaral R, Aguilera-Eguía RA (2019). Relationship between the Human Development Index and its components with oral cancer in Latin America.. J Epidemiol Glob Health.

[5] Weller D, Vedsted P, Rubin G, Walter FM, Emery J, Scott S (2012). The Aarhus statement improving design and reporting of studies on early cancer diagnosis. Br J Cancer.

[6] Güneri P, Epstein JB (2014). Late stage diagnosis of oral cancer components and possible solutions. Oral Oncol.

[7] Stefanuto P, Doucet JC, Robertson C (2014). Delays in treatment of oral cancer a review of the current literature. Oral Surg Oral Med Oral Pathol Oral Radiol.

[8] Alcaraz KI, Wiedt TL, Daniels EC, Yabroff KR, Guerra CE, Wender RC (2020). Understanding and addressing social determinants to advance cancer health equity in the United States a blueprint for practice, research, and policy. CA Cancer J Clin.

[9] Andersen RM, Davidson PL, Andersen RM, Rice TH, Kominski EF (2001). Changing the U.S. health care system: key issues in health services, policy, and management.

[10] Hanna TP, King WD, Thibodeau S, Jalink M, Paulin GA, Harvey-Jones E (2020). Mortality due to cancer treatment delay systematic review and meta-analysis. BMJ.

[11] Tsai WC, Kung PT, Wang YH, Huang KH, Liu SA (2017). Influence of time interval from diagnosis to treatment on survival for oral cavity cancer a nationwide cohort study. PLoS One.

[12] Fujiwara RJ, Judson BL, Yarbrough WG, Husain Z, Mehra S (2017). Treatment delays in oral cavity squamous cell carcinoma and association with survival. Head Neck.

[13] Goel AN, Frangos M, Raghavan G, Sangar S, Lazaro S, Wang MB (2019). Survival impact of treatment delays in surgically managed oropharyngeal cancer and the role of human papillomavirus status. Head Neck.

[14] Murphy CT, Galloway TJ, Handorf EA, Wang L, Mehra R, Flieder DB (2015). Increasing time to treatment initiation for head and neck cancer an analysis of the National Cancer Database. Cancer.

[15] Emerson MA, Golightly YM, Aiello AE, Reeder-Hayes KE, Tan X, Maduekwe U (2020). Breast cancer treatment delays by socioeconomic and health care access latent classes in black and white women. Cancer.

[16] Obrochta CA, Murphy JD, Tsou MH, Thompson CA (2021). Disentangling racial, ethnic, and socioeconomic disparities in treatment for colorectal cancer. Cancer Epidemiol Biomarkers Prev.

[17] Wagle NS, Park S, Washburn D, Ohsfeldt RL, Rich NE, Singal AG (2023). Racial, ethnic, and socioeconomic disparities in treatment delay among patients with hepatocellular carcinoma in the United States.. Clin Gastroenterol Hepatol.

[18] Bajwa MH, Shah MM, Khalid MU, Shamim MS, Baig E, Akhunzada NZ (2022). Time to surgery after radiological diagnosis of brain tumours in Pakistan a nationwide cross-sectional study. J Pak Med Assoc.

[19] Page MJ, McKenzie JE, Bossuyt PM, Boutron I, Hoffmann TC, Mulrow CD (2021). The PRISMA 2020 statement an updated guideline for reporting systematic reviews. BMJ.

[20] Nocini R, Lippi G, Mattiuzzi C (2020). Biological and epidemiologic updates on lip and oral cavity cancers. Ann Cancer Epidemiol.

[21] Ouzzani M, Hammady H, Fedorowicz Z, Elmagarmid A (2016). Rayyan a web and mobile app for systematic reviews. Syst Rev.

[22] Moola S, Munn Z, Tufanaru C, Aromataris E, Sears K, Sfetcu R, Aromataris E, Munn Z JBI Manual for Evidence Synthesis..

[23] Sharma S, Bekelman J, Lin A, Lukens JN, Roman BR, Mitra N (2016). Clinical impact of prolonged diagnosis to treatment interval (DTI) among patients with oropharyngeal squamous cell carcinoma. Oral Oncol.

[24] Morse E, Judson B, Husain Z, Burtness B, Yarbrough WG, Sasaki C (2018). Treatment delays in primarily resected oropharyngeal squamous cell carcinoma national benchmarks and survival associations. Otolaryngol Head Neck Surg.

[25] Morse E, Judson B, Husain Z, Burtness B, Yarbrough W, Sasaki C (2018). National treatment times in oropharyngeal cancer treated with primary radiation or chemoradiation. Oral Oncol.

[26] Su WW, Lee YH, Yen AM, Chen SL, Hsu CY, Chiu SY (2021). Impact of treatment delay on survival of oral/oropharyngeal cancers results of a nationwide screening program. Head Neck.

[27] McKenzie JE, Brennan SE, Higgins JPT, Thomas J, Chandler J, Cumpston M, Li T, Page MJ Cochrane Handbook for Systematic Reviews of Interventions - version 6.4..

[28] Zhang Y, Akl EA, Schünemann HJ (2019). Using systematic reviews in guideline development the GRADE approach. Res Synth Methods.

[29] Rogers SN, Pabla R, McSorley A, Lowe D, Brown JS, Vaughan ED (2007). An assessment of deprivation as a factor in the delays in presentation, diagnosis and treatment in patients with oral and oropharyngeal squamous cell carcinoma. Oral Oncol.

[30] Venchiarutti RL, Clark JR, Palme CE, Shakespare TP, Hill J, Tahir ARM (2020). Influence of remoteness of residence on timeliness of diagnosis and treatment of oral cavity and oropharynx cancer a retrospective cohort study. J Med Imaging Radiat Oncol.

[31] Zhang H, Dziegielewski PT, Jean Nguyen TT, Jeffery CC, O'Connell DA, Harris JR (2015). The effects of geography on survival in patients with oral cavity squamous cell carcinoma.. Oral Oncol.

[32] Massa S, Osazuwa-Peters N, Walker R, Ward G (2018). A national sample of medical expenses for head and neck cancer patients. Int J Radiat Oncol.

[33] Zhao J, Han X, Nogueira L, Fedewa SA, Jemal A, Halpern MT (2022). Health insurance status and cancer stage at diagnosis and survival in the United States. CA Cancer J Clin.

[34] Schoonbeek RC, Zwertbroek J, Plaat BEC, Takes RP, Ridge JA, Strojan P (2021). Determinants of delay and association with outcome in head and neck cancer a systematic review. Eur J Surg Oncol.

[35] Tranby EP, Heaton LJ, Tomar SL, Kelly AL, Fager GL, Backley M (2022). Oral cancer prevalence, mortality, and costs in Medicaid and commercial insurance claims data. Cancer Epidemiol Biomarkers Prev.

[36] Ward E, Jemal A, Cokkinides V, Singh GK, Cardinez C, Ghafoor A (2004). Cancer disparities by race/ethnicity and socioeconomic status. CA Cancer J Clin.

[37] Shin JY, Yoon JK, Shin AK, Diaz AZ (2018). The influence of insurance status on treatment and outcomes in oral cavity cancer an analysis on 46,373 patients. Int J Oral Maxillofac Surg.

[38] van Harten MC, Hoebers FJ, Kross KW, van Werkhoven ED, van den Brekel MW, van Dijk BA (2015). Determinants of treatment waiting times for head and neck cancer in the Netherlands and their relation to survival.. Oral Oncol.

[39] Chen SJ, Kung PT, Huang KH, Wang YH, Tsai WC (2015). Characteristics of the delayed or refusal therapy in breast cancer patients a longitudinal population-based study in Taiwan. PLoS One.

[40] de Mattos Camargo Grossmann S, Sales ACR, Reis DS, Guimarães JC, Silva MT, de Ceno PCG (2021). Knowledge of oral cancer by a Brazilian population.. J Cancer Educ.

[41] González-Ruiz I, Ramos-García P, Ruiz-Ávila I, González-Moles MÁ (2023). Early diagnosis of oral cancer a complex polyhedral problem with a difficult solution. Cancers (Basel).

[42] Matkin L, Ring D (2020). The influence of the United States health care environment and reform on academic medical centers. Hand Clin.

[43] Kain JJ, Birkeland AC, Udayakumar N, Morlandt AB, Stevens TM, Carroll WR (2020). Surgical margins in oral cavity squamous cell carcinoma current practices and future directions. Laryngoscope.

[44] Montagnoli DRABS, Leite VF, Godoy YS, Lafetá VM, Junior EAP, Chaurasia A (2024). Can predictive factors determine the time to treatment initiation for oral and oropharyngeal cancer? A classification and regression tree analysis.. PLoS One.

[45] Daraei P, Moore CE (2015). Racial disparity among the head and neck cancer population. J Cancer Educ.

[46] Kravietz A, Angara P, Le M, Sargi Z (2018). Disparities in screening for head and neck cancer evidence from the NHANES, 2011-2014. Otolaryngol Head Neck Surg.

[47] Nocon CC, Ajmani GS, Bhayani MK (2020). A contemporary analysis of racial disparities in recommended and received treatment for head and neck cancer. Cancer.

[48] Al-Kaabi R, Gamboa AB, Williams D, Marcenes W (2016). Social inequalities in oral cancer literacy in an adult population in a multicultural deprived area of the UK. J Public Health.

[49] Stone JC, Barker TH, Aromataris E, Ritskes-Hoitinga M, Sears K, Klugar M (2023). From critical appraisal to risk of bias assessment clarifying the terminology for study evaluation in JBI systematic reviews. JBI Evid Synth.

