# Supplementary material for: Socioeconomic factors impacting treatment delays in oral and oropharyngeal squamous cell carcinoma: a systematic review
Source: Cad Saude Publica. 2025 Apr 11;41(3):e00121324. doi: 10.1590/0102-311XEN121324 (PMC11996188; doi:10.1590/0102-311XEN121324)
Supplement: Supplementary file 1 [file 1678-4464-csp-41-03-EN121324-s.pdf]

SUPPLEMENTARY MATERIAL

Box S1 Search strategies used in each electronic database (date: from interception to January 2023, updated in December 2023).

**MEDLINE through PubMed**
(((Head and Neck Neoplasms[MeSH Terms]) OR (Mouth Neoplasms[MeSH Terms]) OR (Oropharyngeal Neoplasms[MeSH Terms]) OR (Squamous Cell Carcinoma of Head and Neck[MeSH Terms]) OR ("Head and Neck Neoplasm"[Title/Abstract]) OR ("Cancer of Head and Neck"[Title/Abstract]) OR ("Head and Neck Cancer"[Title/Abstract]) OR ("Head Neoplasms"[Title/Abstract]) OR ("Head Neoplasm"[Title/Abstract]) OR ("Cancer of Head"[Title/Abstract]) OR ("Head Cancers"[Title/Abstract]) OR ("Mouth Neoplasm"[Title/Abstract]) OR ("Oral Neoplasm"[Title/Abstract]) OR ("Oral Neoplasms"[Title/Abstract]) OR ("Cancer of Mouth"[Title/Abstract]) OR ("Mouth Cancers"[Title/Abstract]) OR ("Oral Cancer"[Title/Abstract]) OR ("Oral Cancers"[Title/Abstract]) OR ("Cancer of the Mouth"[Title/Abstract]) OR ("Mouth Cancer"[Title/Abstract]) OR ("Oropharyngeal Neoplasm"[Title/Abstract]) OR ("Oropharynx Neoplasms"[Title/Abstract]) OR ("Oropharynx Neoplasm"[Title/Abstract]) OR ("Oropharyngeal Cancer"[Title/Abstract]) OR ("Oropharyngeal Cancers"[Title/Abstract]) OR ("Oropharynx Cancer"[Title/Abstract]) OR ("Oropharynx Cancers"[Title/Abstract]) OR ("Cancer of the Oropharynx"[Title/Abstract]) OR ("Squamous Cell Carcinoma of the Head and Neck"[Title/Abstract]) OR ("Head and Neck Squamous Cell Carcinoma"[Title/Abstract]) OR ("Oral Tongue Squamous Cell Carcinoma"[Title/Abstract]) OR ("Oral Squamous Cell Carcinoma"[Title/Abstract]) OR ("Oral Cavity Squamous Cell Carcinoma"[Title/Abstract]) OR ("Oral Squamous Cell Carcinomas"[Title/Abstract]) OR ("Squamous Cell Carcinoma of the Mouth"[Title/Abstract]) OR ("Oropharyngeal Squamous Cell Carcinoma"[Title/Abstract])) AND ((Socioeconomic Factors[MeSH Terms]) OR (Income[MeSH Terms]) OR (Poverty[MeSH Terms]) OR (Social Class[MeSH Terms]) OR (Educational Status[MeSH Terms]) OR (Demography[MeSH Terms]) OR ("Socioeconomic Factors") OR ("Educational Status") OR ("Socioeconomic Factor") OR ("Standard of Living") OR ("Living Standard") OR ("Living Standards") OR ("Land Tenure") OR ("Social Inequality") OR ("Social Inequalities") OR ("High-Income Population") OR ("High Income Population") OR ("High-Income Populations") OR ("Incomes") OR ("Savings") OR ("Health Insurances") OR ("Income Distribution") OR ("Income Distributions") OR ("Low-Income Population") OR ("Low-Income Populations") OR ("Low Income Population") OR ("Low Income Populations") OR ("Social Classes") OR ("Socioeconomic Status") OR ("Middle Class Population") OR ("Educational Achievement") OR ("Educational Achievements") OR ("Schooling Level") OR ("Educational Levels") OR ("Demographic") OR ("Population Spatial Distributions") OR ("Demographic Factors") OR ("Demographic Factor") OR ("Demographic Impacts") OR ("Demographic Impact") OR ("Patient Profile")) AND ((Time-to-Treatment[MeSH Terms]) OR ("Time-to-Treatment"[Title/Abstract]) OR (Time-to-Treatments[Title/Abstract]) OR ("Door-to-Treatment Time"[Title/Abstract]) OR ("Door to Treatment Time"[Title/Abstract]) OR ("Delayed Treatment"[Title/Abstract]) OR ("Delayed Treatments"[Title/Abstract]) OR ("Treatment Delay"[Title/Abstract]) OR ("Primary Treatment"[Title/Abstract]) OR ("Waiting Time"[Title/Abstract]) OR ("Timeliness"[Title/Abstract]) OR ("Delay in Treatment"[Title/Abstract]) OR ("Time to Start Treatment"[Title/Abstract]) OR ("Diagnosis-treatment interval"[Title/Abstract]) OR ("Time-to-initiation"[Title/Abstract]) OR ("Treatment initiation"[Title/Abstract]) OR ("Treatment time"[Title/Abstract]) OR ("Treatment times"[Title/Abstract]) OR ("Timely care"[Title/Abstract]) OR ("Diagnosis to treatment"[Title/Abstract]) OR ("Time factors"[Title/Abstract]) OR ("Time to treatment initiation"[Title/Abstract])
**Embase through Elsevier**
("head and neck tumor"/exp OR "head and neck tumor" OR "mouth tumor"/exp OR "mouth tumor" OR "mouth cancer"/exp OR "oropharynx tumor"/exp OR "oropharynx tumor" OR "oropharynx cancer"/exp OR "squamous cell carcinoma of the head and neck"/exp OR "head and neck squamous cell carcinoma"/exp OR "oral tongue squamous cell carcinoma"/exp OR "mouth squamous cell carcinoma"/exp OR "mouth squamous cell carcinoma" OR "oral cavity squamous cell carcinoma"/exp OR "oropharynx squamous cell carcinoma"/exp OR "oropharynx squamous cell carcinoma" OR "mouth carcinoma"/exp OR "mouth carcinoma" OR "oropharynx carcinoma"/exp OR "oropharynx carcinoma" OR "head and neck neoplasm" OR "cancer of head and neck" OR "head and neck cancer" OR "head neoplasms" OR "head neoplasm" OR "cancer of head" OR "head cancers" OR "mouth neoplasm" OR "oral neoplasm" OR "oral neoplasms" OR "cancer of mouth" OR "mouth cancers" OR "oral cancer" OR "oral cancers" OR "cancer of the mouth" OR "mouth cancer" OR "oropharyngeal neoplasm" OR "oropharynx neoplasms" OR "oropharynx neoplasm" OR "cancer of oropharnyx" OR "oropharnyx cancer" OR "oropharnyx cancers" OR "oropharyngeal cancer" OR "oropharyngeal cancers" OR "oropharynx cancer" OR "oropharynx cancers" OR "cancer of the oropharynx" OR "head and neck squamous cell carcinomas" OR "squamous cell carcinoma of the head and neck" OR "head and neck squamous cell carcinoma" OR "oral tongue squamous cell carcinoma" OR "oral squamous cell carcinoma" OR "oral cavity squamous cell carcinoma" OR "oral squamous cell carcinomas" OR "squamous cell carcinoma of the mouth" OR "oropharyngeal squamous cell carcinoma") AND ("socioeconomics"/exp OR "socioeconomics" OR "economic status"/exp OR "economic status" OR "educational status"/exp OR "educational status" OR "income group"/exp OR "income group" OR "poverty"/exp OR "poverty" OR "socioeconomic distribution"/exp OR "socioeconomic distribution" OR "socioeconomic parameters"/exp OR "socioeconomic parameters" OR "socioeconomic vulnerability"/exp OR "socioeconomic vulnerability" OR "household economic status"/exp OR "household economic status" OR "health insurance"/exp OR "health insurance" OR "income" OR "socioeconomic factors" OR "social class" OR "demography" OR "socioeconomic factor" OR "standard of living" OR "living standard" OR "living standards" OR "land tenure" OR "social inequality" OR "social inequalities" OR "high-income population" OR "high income population" OR "high-income populations" OR "incomes" OR "savings" OR "income distribution" OR "income distributions" OR "low-income population" OR "low-income populations" OR "low income population" OR "low income populations" OR "social classes" OR "socioeconomic status" OR "middle class population" OR "educational achievement" OR "educational achievements" OR

“schooling level” OR “educational levels” OR demographic OR “population spatial distributions” OR “demographic factors” OR “demographic factor” OR “demographic impacts” OR “demographic impact” OR “patient profile”) AND (“time to treatment”/exp OR “therapy delay”/exp OR “therapy delay” OR “time to treatment” OR “time-to-treatment” OR “time-to-treatments” OR “door-to-treatment time” OR “door to treatment time” OR “delayed treatment” OR “delayed treatments” OR “treatment delay” OR “primary treatment” OR “waiting time” OR “timeliness” OR “delay in treatment” OR “time to start treatment” OR “diagnosis-treatment interval” OR “time-to-initiation” OR “treatment initiation” OR “treatment time” OR “treatment times” OR “timely care” OR “diagnosis to treatment” OR “time factors” OR “time to treatment initiation”)

Scopus

((TITLE-ABS-KEY ("head and neck tumor") OR TITLE-ABS-KEY ("head neoplasms") OR TITLE-ABS-KEY ("mouth tumor") OR TITLE-ABS-KEY ("mouth cancer") OR TITLE-ABS-KEY ("oropharynx tumor") OR TITLE-ABS-KEY ("oropharynx cancer") OR TITLE-ABS-KEY ("squamous cell carcinoma of the head and neck") OR TITLE-ABS-KEY ("head and neck squamous cell carcinoma") OR TITLE-ABS-KEY ("oral tongue squamous cell carcinoma") OR TITLE-ABS-KEY ("mouth squamous cell carcinoma") OR TITLE-ABS-KEY ("oral cavity squamous cell carcinoma") OR TITLE-ABS-KEY ("oropharynx squamous cell carcinoma") OR TITLE-ABS-KEY ("mouth carcinoma") OR TITLE-ABS-KEY ("oropharynx carcinoma") OR TITLE-ABS-KEY ("Head and Neck Neoplasms") OR TITLE-ABS-KEY ("Mouth Neoplasms") OR TITLE-ABS-KEY ("Oropharyngeal Neoplasms") OR TITLE-ABS-KEY ("Squamous Cell Carcinoma of Head and Neck") OR TITLE-ABS-KEY ("Oral Neoplasm") OR TITLE-ABS-KEY ("Oral Cancer") OR TITLE-ABS-KEY ("Oropharyngeal Cancer") OR TITLE-ABS-KEY ("Oral Squamous Cell Carcinoma") OR TITLE-ABS-KEY ("Oral Cavity Squamous Cell Carcinoma") OR TITLE-ABS-KEY ("Oral Squamous Cell Carcinomas") OR TITLE-ABS-KEY ("Squamous Cell Carcinoma of the Mouth") OR TITLE-ABS-KEY ("Oropharyngeal Squamous Cell Carcinoma"))) AND ((ALL (socioeconomics) OR ALL ("economic status") OR ALL ("educational status") OR ALL ("income group") OR ALL (poverty) OR ALL ("socioeconomic distribution") OR ALL ("socioeconomic parameters") OR ALL ("socioeconomic vulnerability") OR ALL ("household economic status") OR ALL ("health insurance") OR ALL ("Socioeconomic Factors") OR ALL ("Social Class") OR ALL (demography) OR ALL ("Standard of Living") OR ALL ("Land Tenure") OR ALL ("Social Inequality") OR ALL ("High-Income Population") OR ALL ("Health Insurances") OR ALL ("Income Distribution") OR ALL ("Low-Income Population") OR ALL ("Middle Class Population") OR ALL ("Schooling Level") OR ALL ("Demographic Impacts") OR TITLE-ABS-KEY ("Patient Profile"))) AND ((TITLE-ABS-KEY ("time to treatment") OR TITLE-ABS-KEY ("Therapy delay") OR TITLE-ABS-KEY ("Time-to-Treatment") OR TITLE-ABS-KEY ("Door-to-Treatment Time") OR TITLE-ABS-KEY ("Door to Treatment Time") OR TITLE-ABS-KEY ("Delayed Treatment") OR TITLE-ABS-KEY ("Delayed Treatments") OR TITLE-ABS-KEY ("Treatment Delay") OR TITLE-ABS-KEY ("Primary Treatment") OR TITLE-ABS-KEY ("Waiting Time") OR TITLE-ABS-KEY (timeliness) OR TITLE-ABS-KEY ("Delay in Treatment") OR TITLE-ABS-KEY ("Time to Start Treatment") OR TITLE-ABS-KEY ("Diagnosis-treatment interval") OR TITLE-ABS-KEY ("Time-to-initiation") OR TITLE-ABS-KEY ("Treatment initiation") OR TITLE-ABS-KEY ("Treatment time") OR TITLE-ABS-KEY ("Treatment times") OR TITLE-ABS-KEY ("Timely care") OR TITLE-ABS-KEY ("Diagnosis to treatment") OR TITLE-ABS-KEY ("Time factors") OR TITLE-ABS-KEY ("Time to treatment initiation")))

Web of science

TS=((“head and neck tumor” OR “head and neck tumor” OR “mouth tumor” OR “mouth tumor” OR “mouth cancer” OR “oropharynx tumor” OR “oropharynx tumor” OR “oropharynx cancer” OR “squamous cell carcinoma of the head and neck” OR “head and neck squamous cell carcinoma” OR “oral tongue squamous cell carcinoma” OR “mouth squamous cell carcinoma” OR “mouth squamous cell carcinoma” OR “oral cavity squamous cell carcinoma” OR “oropharynx squamous cell carcinoma” OR “oropharynx squamous cell carcinoma” OR “mouth carcinoma” OR “mouth carcinoma” OR “oropharynx carcinoma” OR “oropharynx carcinoma” OR “head and neck neoplasm” OR “cancer of head and neck” OR “head and neck cancer” OR “head neoplasms” OR “head neoplasm” OR “cancer of head” OR “head cancers” OR “mouth neoplasm” OR “oral neoplasm” OR “oral neoplasms” OR “cancer of mouth” OR “mouth cancers” OR “oral cancer” OR “oral cancers” OR “cancer of the mouth” OR “mouth cancer” OR “oropharyngeal neoplasm” OR “oropharynx neoplasms” OR “oropharynx neoplasm” OR “cancer of oropharnyx” OR “oropharnyx cancer” OR “oropharnyx cancers” OR “oropharyngeal cancer” OR “oropharyngeal cancers” OR “oropharynx cancer” OR “oropharynx cancers” OR “cancer of the oropharynx” OR “head and neck squamous cell carcinomas” OR “squamous cell carcinoma of the head and neck” OR “head and neck squamous cell carcinoma” OR “oral tongue squamous cell carcinoma” OR “oral squamous cell carcinoma” OR “oral cavity squamous cell carcinoma” OR “oral squamous cell carcinomas” OR “squamous cell carcinoma of the mouth” OR “oropharyngeal squamous cell carcinoma”) AND (“socioeconomics” OR “socioeconomics” OR “economic status” OR “economic status” OR “educational status” OR “educational status” OR “income group” OR “income group” OR “poverty” OR “poverty” OR “socioeconomic distribution” OR “socioeconomic distribution” OR “socioeconomic parameters” OR “socioeconomic parameters” OR “socioeconomic vulnerability” OR “socioeconomic vulnerability” OR “household economic status” OR “household economic status” OR “health insurance” OR “health insurance” OR “income” OR “socioeconomic factors” OR “social class” OR “demography” OR “socioeconomic factor” OR “standard of living” OR “living standard” OR “living standards” OR “land tenure” OR “social inequality” OR “social inequalities” OR “high-income population” OR “high income population” OR “high-income populations” OR “incomes” OR “savings” OR “income distribution” OR “income distributions” OR “low-income population” OR “low-income populations” OR “low income population” OR “low income populations” OR “social classes” OR “socioeconomic status” OR “middle class population” OR “educational achievement” OR “educational achievements” OR “schooling level” OR “educational levels” OR demographic OR “population spatial distributions” OR “demographic factors” OR “demographic factor” OR “demographic impacts” OR “demographic impact” OR “patient profile”) AND (“time to treatment” OR “therapy delay” OR “therapy delay” OR “time to treatment” OR “time-to-treatment” OR “time-to-

treatments" OR "door-to-treatment time" OR "door to treatment time" OR "delayed treatment" OR "delayed treatments" OR "treatment delay" OR "primary treatment" OR "waiting time" OR "timeliness" OR "delay in treatment" OR "time to start treatment" OR "diagnosis-treatment interval" OR "time-to-initiation" OR "treatment initiation" OR "treatment time" OR "treatment times" OR "timely care" OR "diagnosis to treatment" OR "time factors" OR "time to treatment initiation"))
**Virtual Health Library through BIREME**<br><br>((mh:(head AND neck neoplasms)) OR (mh:(mouth neoplasms)) OR (mh:(oropharyngeal neoplasms)) OR (mh:(squamous cell carcinoma of head AND neck)) OR (cancer of head) OR (cancer of head AND neck) OR (cancer of the head) OR (cancer of the head AND neck) OR (head cancer) OR (head cancers) OR (head neoplasm) OR (head neoplasms) OR (head AND neck cancer) OR (head AND neck neoplasm) OR (cancer of mouth) OR (cancer of the mouth) OR (mouth cancer) OR (mouth cancers) OR (mouth neoplasm) OR (oral cancer) OR (oral cancers) OR (oral neoplasm) OR (oral neoplasms) OR (cancer of oropharynx) OR (cancer of the oropharynx) OR (oropharnyx cancer) OR (oropharnyx cancers) OR (oropharyngeal cancer) OR (oropharyngeal cancers) OR (oropharyngeal neoplasm) OR (oropharynx cancer) OR (oropharynx cancers) OR (oropharynx neoplasm) OR (oropharynx neoplasms) OR (head AND neck squamous cell carcinomas) OR (head AND neck squamous cell carcinoma) OR (oral cavity squamous cell carcinoma) OR (oral squamous cell carcinoma) OR (oral squamous cell carcinomas) OR (oral tongue squamous cell carcinoma) OR (oropharyngeal squamous cell carcinoma) OR (squamous cell carcinoma of the mouth)) AND ((mh:(socioeconomic factors)) OR (high income population) OR (high-income population) OR (high-income populations) OR (land tenure) OR (living standard) OR (living standards) OR (social inequalities) OR (social inequality) OR (social inequity) OR (socioeconomic factor) OR (standard of living) OR (educational level) OR (educational status) OR (social class)) AND ((mh:(time-to-treatment)) OR (delayed treatment) OR (delayed treatments) OR (door TO treatment time) OR (time TO treatment) OR (time TO treatments) OR (time-to-treatments) OR (treatment delay) OR (treatment delays) OR (primary treatment) OR (waiting time) OR (timeliness) OR (time TO start treatment) OR (diagnosis-treatment interval) OR (time-to-initiation) OR (treatment initiation) OR (treatment time) OR (treatment times))
**Google Scholar**<br><br>("mouth neoplasms" OR "oropharyngeal neoplasms" OR "oral squamous cell carcinoma") AND ("socioeconomic factors" OR income OR "educational level") AND ("time-to-treatment" OR "treatment delay" OR timeless OR "waiting time")
**OpenGrey:**<br><br>Oral cancer AND treatment delay

Box S2 References of excluded studies from the systematic review and reason for exclusion.

	Study	Reason for exclusion
**1.**	Abdo EN, Garrocho Ade A, Barbosa AA, Oliveira EL, Franca-Filho L, Negri SL, Pordeus IA. Time elapsed between the first symptoms, diagnosis and treatment of oral cancer patients in Belo Horizonte, Brazil. Med Oral Patol Oral Cir Bucal. 2007 Nov 1;12(7):E469-73.	Professional delay (interval between symptoms to diagnosis).
**2.**	Agarwal AK, Sethi A, Sareen D, Dhingra S. Treatment delay in oral and oropharyngeal cancer in our population: the role of socioeconomic factors and health-seeking behaviour. Indian J Otolaryngol Head Neck Surg. 2011 Apr;63(2):145-50. doi: 10.1007/s12070-011-0134-9.	Patient delay (interval between self-perception of symptoms and first consultation).
**3.**	Agarwal P, Agrawal RR, Jones EA, Devaiah AK. Social Determinants of Health and Oral Cavity Cancer Treatment and Survival: A Competing Risk Analysis. Laryngoscope. 2020 Sep;130(9):2160-2165.	The study compares socioeconomic factors related to survival. It does not report time-to-treatment.
**4.**	Amsbaugh MJ, Yusuf M, Cash E, Silverman C, Potts K, Dunlap N. Effect of time to simulation and treatment for patients with oropharyngeal cancer receiving definitive radiotherapy in the era of risk stratification using smoking and human papillomavirus status. Head Neck. 2018 Apr;40(4):687-695.	The study is about time-to-treatment. It does not report the socioeconomic factors of the sample.
**5.**	Andrews GA, Patel VA, Derr J, Zhu J. Timeliness of Treatment for Head and Neck Squamous Cell Carcinoma. Head and Neck Surgery. Otolaryngol Head Neck Surger. 2015;153(1_suppl):P55–P79.	Abstract.
**6.**	Arbes SJ Jr, Olshan AF, Caplan DJ, Schoenbach VJ, Slade GD, Symons MJ. Factors contributing to the poorer survival of black Americans diagnosed with oral cancer (United States). Cancer Causes Control. 1999; 10(6):513-23.	The study compares socioeconomic factors related to survival. It does not report time-to-treatment.
**7.**	Baker S, Banerjee R, Debenham B. EP-1056: Treatment delays are associated with disease upstaging in oropharyngeal squamous cell carcinoma. Radiotherapy and Oncology. 2016; 119: S510.	Abstract.
**8.**	Balchander D. et al. Prognostic Significance of Referral Patterns and Time Trends in Head and Neck Cancer Management. Int J Radiat Oncol Biol Phys. 2022; 112(5):e27.	Abstract.
**9.**	Balk M, Rupp R, Craveiro AV, Allner M, Grundtner P, Eckstein M, Hecht M, Iro H, Gostian AO. The COVID-19 pandemic and its consequences for the diagnosis and therapy of head and neck malignancies. Eur Rev Med Pharmacol Sci. 2022 Jan;26(1):284-290.	The study is about head and neck cancer patient delay in COVID-19 pandemic.
**10.**	Barros-Silva PG, Fontes-Borges MM, Costa-Dias C, Mota-Lemos JV, Socorro-Saldanha-Cunha MD, Fernandes-Souza E, Sousa-Dantas T, Bitu-Sousa F. Clinical-pathological and sociodemographic factors associated with the distant metastasis and overall survival of oral cavity and oropharynx squamous cell carcinoma. Med Oral Patol Oral Cir Bucal. 2020 May 1;25(3):e375-e382.	The study compares socioeconomic factors related to survival. It does not report time-to-treatment.
**11.**	Beaudoin PL, Munden J, Faye M, Ndiaye IC, Sewitch M, Ayad T, Poenaru D. Identifying Barriers in Access to Care for Head and Neck Cancer Patients: A Field Study in Dakar. Laryngoscope. 2022; 132(6):1219-1223.	Professional delay (interval between symptoms to diagnosis).
**12.**	Bedir A, Abera SF, Efremov L, Hassan L, Vordermark D, Medenwald D. Socioeconomic disparities in head and neck cancer survival in Germany: a causal mediation analysis using population-based cancer registry data. J Cancer Res Clin Oncol. 2021;147(5):1325-1334.	The study compares socioeconomic factors related to survival. It does not report time-to-treatment.
**13.**	Bhattacharyya N, Abemayor E. Patterns of hospital utilization for head and neck cancer care: changing demographics. JAMA Otolaryngol Head Neck Surg. 2015 Apr;141(4):307-12.	The study compares socioeconomic factors related to survival. It does not report time-to-treatment.
**14.**	Borrayo EA, Scott KL, Drennen AR, MacDonald T, Nguyen J. Determinants of Treatment Delays Among Underserved Hispanics With Lung and Head and Neck Cancers. Cancer Control. 2016; 23(4):390-400.	Head and neck patient delay without separating oral cancer in the analysis.
**15.**	Brinkerhoff BT, Choong NW, Massey BL, Gore EM, Firat SY, Wong SJ, Campbell BH, Schapira RM, Visotcky AM, Tripp PM. Diagnosis to Treatment Interval and Outcome in Patients with LocallyAdvanced Squamous Cell Carcinoma of the Head and Neck in a Veterans Affairs Medical Center. J Cancer Sci Ther. 2012; 4(5):111-15.	Head and neck patient delay without separating oral cancer in the analysis.
**16.**	Brocklehurst P, Rafiq R, Lowe D, Rogers S. Analysis of the impact of deprivation on urgent suspected head and neck cancer referrals in the Mersey region between January 2004 to December 2006. Br J Oral Maxillofac Surg.	Professional delay (interval between symptoms and diagnosis).

	2012; 50(3):215-20.	
**17.**	Carey RM, Fathy R, Shah RR, Rajasekaran K, Cannady SB, Newman JG, Ibrahim SA, Brant JA. Association of Type of Treatment Facility With Overall Survival After a Diagnosis of Head and Neck Cancer. JAMA Netw Open. 2020; 3(1):e1919697.	The study is not about time to treatment. It reports on type of treatment facility and survival.
**18.**	Carlsen AH, Eriksen JG, Godballe C, Johansen J, Sørensen JA, Bjørndal K. Impact of age, comorbidity, and WHO performance status on delay of treatment in patients undergoing fast-track work-up for head and neck cancer. J Geriatr Oncol. 2019; 10(2):259-264.	Professional delay (interval between symptoms and diagnosis).
**19.**	Carroll WR, Kohler CL, Carter VL, Hannon L, Skipper JB, Rosenthal EL. 2009. Barriers to early detection and treatment of head and neck squamous cell carcinoma in African American men. Head Neck. 2009; 31(12):1557-62.	Head and neck patient delay without separating oral cancer in the analysis.
**20.**	Caudell JJ, Locher JL, Bonner JA. Diagnosis-to-treatment interval and control of locoregionally advanced head and neck cancer. Arch Otolaryngol Head Neck Surg. 2011; 137(3):282-5.	Head and neck patient delay without separating oral cancer in the analysis.
**21.**	Chandarana MN, Pai PS. Demography and treatment pattern of patients with head and neck carcinoma presenting to a tertiary care center in India: Need for urgent decentralization of cancer care. South Asian J Cancer. 2020; 9(1):38-42.	The study is not about time to treatment.
**22.**	Chapman CH, Ryan WR, Yom SS. Prevalence of Highly Self-Directed Care Among Patients with HPV-Associated Oropharyngeal Cancer. Int J Radiat Oncol Biol Phys. 2017; 99(2): E327.	Abstract.
**23.**	Chen AY, Schrag N, Hao Y, Stewart A, Ward E. Changes in Treatment of Advanced Oropharyngeal Cancer, 1985-2001. Laryngoscope. 2007; 117(1):16–21.	The study is about socioeconomic factors related to treatment facility types. It does not report on time-to-treatment.
**24.**	Chen AY, Zhu J, Fedewa S. Temporal trends in oropharyngeal cancer treatment and survival: 1998-2009. Laryngoscope. 2013; 124(1): 131–138.	The study is about types of oropharyngeal cancer treatment over the years. It does not report on time-to-treatment.
**25.**	Chiliti BA, Campos WG, Gallo CB, Lemos CA. Oral cancer analysis in a Brazilian city: interval between diagnosis and treatment. Braz Oral Res. 2022; 36: e073.	The study is no related to Squamous Cell Carcinoma.
**26.**	Chu KP, Habbous S, Kuang Q, Boyd K, Mirshams M, Liu F-F, … Liu G. Socioeconomic status, human papillomavirus, and overall survival in head and neck squamous cell carcinomas in Toronto, Canada. Cancer Epidemiol. 2016; 40: 102–112.	The study describes socioeconomic factors related to survival. It does not report time-to-treatment.
**27.**	Cook L, Woods C, Nicholls T, Ooi EH. Delays in Time to Head and Neck Cancer Treatment: A South Australian Perspective. Medicina (Kaunas). 2022; 58(2):145.	The study includes other types of cancer, not just Squamous Cell Carcinoma.
**28.**	Costa AASD, Caldeira PC, Sousa AA, Tibúrcio JD, Belligoli LQG, Santos VBD, Bretas PMC, Nunes LL, Prado Neto SCD, Silva GW, Soares JMA. Oral and oropharyngeal cancer: time from first symptoms to treatment initiation and associated factors. Braz Oral Res. 2023 May 29;37:e054.	The study not report time-to-treatment according to the objective of this review.
**29.**	da Conceição MGD, Emmerick ICM, Figueiró AC, Luiza VL. Oral cancer patient's profile and time to treatment initiation in the public health system in Rio de Janeiro, Brazil. BMC Health Serv Res. 2021; 21(1):145.	Patient delay (interval between first symptom and diagnosis).
**30.**	Dantas TS, de Barros Silva PG, Sousa EF, da Cunha MDP, de Aguiar ASW, Costa FWG, Mota MRL, Alves APNN, Sousa FB. Influence of Educational Level, Stage, and Histological Type on Survival of Oral Cancer in a Brazilian Population: A Retrospective Study of 10 Years Observation. Medicine (Baltimore). 2016; 95(3):e2314.	The study describes socioeconomic factors related to survival. It does not report time-to-treatment.
**31.**	Davis GE, Bryson CL, Yueh B, McDonell MB, Micek MA, Fihn SD. Treatment delay associated with alternative medicine use among veterans with head and neck cancer. Head Neck. 2006; 28(10):926-31.	Head and neck patient delay without separating oral cancer in the analysis.
**32.**	Dayan G, Bahig H, Fortin B, Filion É, Nguyen-Tan PF, O'Sullivan B, Charpentier D, Soulières D, Gologan O, Nelson K, Létourneau L, Schmittbuhl M, Ayad T, Bissada E, Guertin L, Tabet P, Christopoulos A. Predictors of prolonged treatment time intervals in oral cavity cancer. Oral Oncol. 2023;147:106622.	The study is about time-to-treatment. It does not report the socioeconomic factors of the sample.

**33.**	De Luca P, Bisogno A, Colacurcio V, Marra P, Cassandro C, Camaioni A, Cassandro E, Scarpa A. Diagnosis and treatment delay of head and neck cancers during COVID-19 era in a tertiary care academic hospital: what should we expect? Eur Arch Otorhinolaryngol. 2022; 279(2):961-965.	Head and neck patient delay due to COVID-19 pandemic.
**34.**	Diz Dios P, Padrón González N, Seoane Lestón J, Tomás Carmona I, Limeres Posse J, Varela-Centelles P. "Scheduling delay" in oral cancer diagnosis: a new protagonist. Oral Oncol. 2005; 41(2):142-6.	The study is about scheduling delay, not treatment delay.
**35.**	Dooley S, Khakoo NS, Perlow H, Kwon D, Nicolli E, Yechieli R, Samuels MA, Mora M, Friedman LM, Samuels S. Improved Care for Patients Evaluated in a Head and Neck Multidisciplinary Clinic at a Safety Net Hospital. Int J Radiat Oncol Biol Phys. 2020; 108(3): E817-E818.	Abstract.
**36.**	Dost P, Talanow DD, Kaiser S, Hirche H, Jahnke K. Zum Zeitintervall zwischen Symptom- und Behandlungsbeginn bei Kopf- und Halstumoren [The time span between symptom onset and starting treatment in head and neck tumors]. HNO. 1996; 44(9):492-6. German.	Patient delay (interval between first symptom and diagnosis).
**37.**	Elkins W, Mbah O, Benson JT, Farnan L, Padilla N, Cykert S, Reeve BB, Corbie-Smith G, Samuel CA. Abstract D071: Impact of racial differences in financial burden on time to treatment. Cancer Epidemiol Biomarkers Prev. 2020; 29 (6_Supplement_2): D071.	Abstract.
**38.**	Esmaelbeigi F, Hadji M, Harirchi I, Omranipour R, vand Rajabpour M, Zendehdel K. Factors affecting professional delay in diagnosis and treatment of oral cancer in Iran. Arch Iran Med. 2014; 17(4):253-7.	Professional delay (interval between symptoms and diagnosis).
**39.**	Felippu AW, Freire EC, Silva Rde A, Guimarães AV, Dedivitis RA. Impact of delay in the diagnosis and treatment of head and neck cancer. Braz J Otorhinolaryngol. 2016; 82(2):140-3.	Head and neck patient delay without separating oral cancer in the analysis.
**40.**	Flukes S, Garry S, Hinton-Bayre A, Lindsay A. Pre-treatment wait time for head and neck cancer patients in Western Australia: description of a new metric and examination of predictive factors. ANZ J Surg. 2019; 89(7-8):858-862.	Head and neck patient delay without separating oral cancer in the analysis.
**41.**	Fortin A, Bairati I, Albert M, Moore L, Allard J, Couture C. Effect of treatment delay on outcome of patients with early-stage head-and-neck carcinoma receiving radical radiotherapy. Int J Radiat Oncol Biol Phys. 2002; 52(4):929-36.	The delay was defined as the time from initial evaluation by a radiation oncologist to the start of radiotherapy.
**42.**	Gao J, Tseng C, Barinsky GL, Varughese D, Baredes S, Park RC. Characterizing the effects of population density on survival for salivary gland malignancies. Otolaryngol- Head Neck Sur. 2020; 163(1):P179.	Abstract.
**43.**	Guizard AV, Dejardin O, Launay L, Bara S, Lapôtre-Ledoux B, Babin E, Launoy G, Ligier K. What are the real waiting times for therapeutic management of head and neck cancer: a study in the general population in the north-west of France. Eur Arch Otorhinolaryngol. 2016; 273(11):3951-3958.	Head and neck patient delay without separating oral cancer in the analysis.
**44.**	Grønhøj C, Jensen D, Dehlendorff C, Nørregaard C, Andersen E, Specht L, Charabi B, von Buchwald C. Impact of Time to Treatment Initiation in Patients with Human Papillomavirus-positive and -negative Oropharyngeal Squamous Cell Carcinoma. Clin Oncol (R Coll Radiol). 2018; 30(6):375-381.	The study is about time intervals from diagnosis to treatment. It does not report the socioeconomic factors of the sample.
**45.**	Hemmi T, Yusa K, Kasuya S, Yamanouchi H, Ishikawa S, Kobayashi T, Iino M. Influence of interval between biopsy and surgery on prognosis of patients with early-stage oral squamous cell carcinoma: A preliminary study. J Oral Maxil Sur Med Pathol. 2019; 31(3):159-162.	The study is about time intervals from diagnosis to treatment. It does not report the socioeconomic factors of the sample.
**46.**	Ho AS, Kim S, Tighiouart M, Mita A, Scher KS, Epstein JB, Laury A, Prasad R, Ali N, Patio C, Clair JM, Zumsteg ZS. Quantitative survival impact of composite treatment delays in head and neck cancer. Cancer. 2018; 124(15):3154-3162.	Head and neck patient delay without separating oral cancer in the analysis.
**47.**	Hollows P, McAndrew PG, Perini MG. 2000. Delays in the referral and treatment of oral squamous cell carcinoma. Br Dent J. 188(5):262-5.	Professional delay (time to referral to treatment).
**48.**	Housri N, Kim S, Haffty BG, Gabel M. African Americans Face Significant Delays to Treatment After Diagnosis of Oropharyngeal Carcinoma. Int J Radiat Oncol Biol Phys. 2012; 84(3):S469.	Abstract.
**49.**	Hsiehchen D, Muquith M, Haque W, Espinoza M, Yopp A, Beg MS. Clinical Efficiency and Safety Outcomes of Virtual Care for Oncology Patients During the COVID-19 Pandemic. JCO Oncol Pract. 2021; 17(9):e1327-e1332.	The study is about different types of cancer.

**50.**	Itamura K, Kokot N, Sinha U, Swanson M. Association of insurance type with time course of care in head and neck cancer management. Laryngoscope. 2020; 130(11):E587-E592.	Head and neck patient delay without separating oral cancer in the analysis.
**51.**	Iyer HS, Kohler RE, Ramogola-Masire D, Brown C, Molebatsi K, Grover S, Kablay I, Bvochora-Nsingo M, Efstathiou JA, Lockman S, Tapela N, Dryden-Peterson SL. Explaining disparities in oncology health systems delays and stage at diagnosis between men and women in Botswana: A cohort study. PLoS One. 2019 Jun 6;14(6):e0218094.	The study includes overall types of cancer, including head and neck cancer.
**52.**	Jafari A, Najafi Sh, Moradi F, Kharazifard M, Khami M. Delay in the diagnosis and treatment of oral cancer. J Dent (Shiraz). 2013; 14(3):146-50.	Professional delay (delay in diagnosis impacts on delay in treatment).
**53.**	Jensen AR, Nellemann HM, Overgaard J. Tumor progression in waiting time for radiotherapy in head and neck cancer. Rad Oncol. 2007; 84(1):5-10.	Head and neck patient delay without separating oral cancer in the analysis.
**54.**	Jensen JS, Jakobsen KK, Mirian C, Ghanizada M, Håkansson K, Wessel I, Grønhøj C, Rasmussen JH, von Buchwald C. Impact of time to treatment initiation for patients with oral cavity squamous cell carcinoma: a population-based, retrospective study. Acta Oncol. 2021; 60(4):491-496.	The study is about time intervals from diagnosis to treatment. It does not report the socioeconomic factors of the sample.
**55.**	Jin MC, Harris JP, Sabolch AN, Gensheimer M, Le QT, Beadle BM, Pollom EL. Proton radiotherapy and treatment delay in head and neck squamous cell carcinoma. Laryngoscope. 2020; 130(11):E598-E604.	Head and neck patient delay without separating oral cancer in the analysis.
**56.**	Joshi P, Nair S, Chaturvedi P, Nair D, Agarwal JP, D'Cruz AK. Delay in seeking specialized care for oral cancers: experience from a tertiary cancer center. Indian J Cancer. 2014; 51(2):95-7.	Professional delay (delay in diagnosis impacts on treatment delay).
**57.**	Kato I, Neale AV. Does use of alternative medicine delay treatment of head and neck cancer? A surveillance, epidemiology, and end results (SEER) cancer registry study. Head Neck. . 2008; 30(4):446-54.	Head and neck patient delay without separating oral cancer in the analysis.
**58.**	Kompelli A, Cartmell KB, Sterba KR, Alberg AJ, Xiao CC, Sood AJ, Garrett-Mayer E, White-Gilbertson SJ, Rosenzweig SA, Day TA. An assessment of racial differences in epidemiological, clinical and psychosocial factors among head and neck cancer patients at the time of surgery. World J Otorhinolaryngol Head Neck Surg. 2020; 6(1):41-48.	Head and neck patient delay without separating oral cancer in the analysis.
**59.**	Kowalski LP, Carvalho AL. Influence of time delay and clinical upstaging in the prognosis of head and neck cancer. Oral Oncol. 2001 Jan;37(1):94-8.	Head and neck patient delay without separating oral cancer in the analysis.
**60.**	Krishnatreya M, Kataki AC, Sharma JD, Nandy P, Rahman T, Kumar M, Gogoi G, Hoque N. Educational levels and delays in start of treatment for head and neck cancers in North-East India. Asian Pac J Cancer Prev. 2014; 15(24):10867-9.	Head and neck patient delay without separating oral cancer in the analysis.
**61.**	Kumar A, Sahu SK, Karunanithi G, Laksham KB. Treatment Seeking Behavior, Treatment Cost and Quality of Life of Head and Neck Cancer Patients: A Cross-Sectional Analytical Study from South India. Asian Pac J Cancer Prev. 2021; 22(9):3023-3030.	Head and neck patient delay without separating oral cancer in the analysis.
**62.**	Lai DW, Kim J, Marciscano A, Buckley SA, Schmidt BL, Cohen RF, Nierodzik MNR, Myssiorek D, DeLacure MD, Sanfilippo N, Seetharamu N. The impact of interventions on provider and treatment delays in head and neck cancer patients. J Clinical Oncol. 2012; 30(15): e16052-e16052.	Abstract.
**63.**	Leachman BK, DeMora L, Churilla TM, Ebersole B, Bauman J, Ridge JA, Lango M, Egleston B, Galloway TJ. Multidisciplinary Care of Head and Neck Cancer in Elderly Patients. Int J Rad Oncol Biol Phys. 2018; 100(5):1329.	Abstract.
**64.**	Lee SC, Tang IP, Avatar SP, Ahmad N, Selva KS, Tay KK, Vikneswaran T, Tan TY. Head and neck cancer: possible causes for delay in diagnosis and treatment. Med J Malaysia. 2011; 66(2):101-4.	Head and neck patient delay without separating oral cancer in the analysis.
**65.**	Lee YH. Impact of Delay in Treatment on Survival of 6353 Oral Cancers: Results of a Nationwide Screening Program in Taiwan. J Global Oncol. 2018; 4(Supplement 2): 204s-204s.	Abstract.
**66.**	Le Tourneau C, Jung GM, Borel C, Bronner G, Flesch H, Velten M. Prognostic factors of survival in head and neck cancer patients treated with surgery and postoperative radiation therapy. Acta Otolaryngol. 2008; 128(6):706-12.	Head and neck patient delay without separating oral cancer in the analysis.
**67.**	Liao CT, Chen HN, Wen YW, Lee SR, Ng SH, Liu TW, Tsai ST, Tsai MH, Lin JC, Lou PJ, Wang CP, Chu PY, Leu YS, Tsai KY, Terng SD, Chen TM, Wang CH, Chien CY, Chen WC, Lee LY, Lin CY, Wang HM, Lin CH, Fang TJ,	The study is about time-to-treatment. It does not report the socioeconomic factors of the sample.

	Huang SF, Kang CJ, Chang KP, Yang LY, Yen TC. Association between the diagnosis-to-treatment interval and overall survival in Taiwanese patients with oral cavity squamous cell carcinoma. Eur J Cancer. 2017; 72:226-234.	
**68.**	Liao DZ, Schlecht NF, Rosenblatt G, Kinkhabwala CM, Leonard JA, Ference RS, Prystowsky MB, Ow TJ, Schiff BA, Smith RV, Mehta V. Association of Delayed Time to Treatment Initiation With Overall Survival and Recurrence Among Patients With Head and Neck Squamous Cell Carcinoma in an Underserved Urban Population. JAMA Otolaryngol Head Neck Surg. 2019; 145(11):1001-1009.	The study is about time-to-treatment. It does not report socioeconomic factors of the sample.
**69.**	Liaquat A, Naseer R, Rashid M, Khan SM, Haq EU, Tayyab TF. The Role of Socioeconomic Status and Literacy in the treatment delay in Oral Cancer patients Visiting Mayo Hospital Lahore, Pakistan – A Tertiary Care Hospital. Pak J Med Health Sci. 2021;15(8): 1785-87.	Patient delay (interval of self-perception of the first symptoms impacts treatment delay).
**70.**	Lopez-Cedrún JL, Varela-Centelles P, Otero-Rico A, Vázquez-Mahía I, Seoane J, Castelo-Baz P, Seoane-Romero J. Overall time interval ("Total diagnostic delay") and mortality in symptomatic oral cancer: A U-shaped association. Oral Oncol. 2020; 104:104626.	Patient and Professional delay (interval between first symptoms and treatment).
**71.**	Lyhne NM, Christensen A, Alanin MC, Bruun MT, Jung TH, Bruhn MA, Jespersen JB, Kristensen CA, Andersen E, Godballe C, Buchwald C, Bundgaard T, Johansen J, Lambertsen K, Primdahl H, Toustrup K, Sørensen JA, Overgaard J, Grau C. Waiting times for diagnosis and treatment of head and neck cancer in Denmark in 2010 compared to 1992 and 2002. Eur J Cancer. 2013; 49(7):1627-33.	Head and neck patient delay without separating oral cancer in the analysis.
**72.**	McDermott JD, Eguchi M, Morgan R, Amini A, Goddard JA, Borrayo EA, Karam SD. Elderly Black Non-Hispanic Patients With Head and Neck Squamous Cell Cancer Have the Worst Survival Outcomes. J Natl Compr Canc Netw. 2020; 19(1):57-67.	Head and neck patient delay without separating oral cancer in the analysis.
**73.**	Metzger K, Moratin J, Horn D, Pilz M, Ristow O, Hoffmann J, Freier K, Engel M, Freudlsperger C. Treatment delay in early-stage oral squamous cell carcinoma and its relation to survival. J Craniomaxillofac Surg. 2021; 49(6):462-467.	Patient delay (treatment delay was defined between the initial presentation of the patient and the date of surgery).
**74.**	Metzger K, Mrosek J, Zittel S, Pilz M, Held T, Adeberg S, Ristow O, Hoffmann J, Engel M, Freudlsperger C, Moratin J. Treatment delay and tumor size in patients with oral cancer during the first year of the COVID-19 pandemic. Head Neck. 2021; 43(11):3493-3497.	Patient delay (treatment delay was defined between the initial presentation of the patient and the date of surgery).
**75.**	Molina MA, Cheung MC, Perez EA, Byrne MM, Franceschi D, Moffat FL, Livingstone AS, Goodwin WJ, Gutierrez JC, Koniaris LG. African American and poor patients have a dramatically worse prognosis for head and neck cancer: an examination of 20,915 patients. Cancer. 2008; 113(10):2797-806.	The study is not about time-to-treatment but only about socioeconomic factors.
**76.**	Morse E, Fujiwara RJT, Judson B, Mehra S. Treatment Times in Salivary Gland Cancer: National Patterns and Association with Survival. Otolaryngol Head Neck Surg. 2018; 159(2):283-292.	The study is not about only Squamous Cell Carcinoma.
**77.**	Murphy CT, Galloway TJ, Handorf EA, Egleston BL, Wang LS, Mehra R, Flieder DB, Ridge JA. Survival Impact of Increasing Time to Treatment Initiation for Patients With Head and Neck Cancer in the United States. J Clin Oncol. 2016; 34(2):169-78.	Head and neck patient delay without separating oral cancer in the analysis.
**78.**	Murphy CT, Galloway TJ, Handorf EA, Wang L, Mehra R, Flieder DB, Ridge JA. Increasing time to treatment initiation for head and neck cancer: an analysis of the National Cancer Database. Cancer. 2015; 121(8):1204-13.	Head and neck patient delay without separating oral cancer in the analysis.
**79.**	Naghavi AO, Demetriou SK, Venkat PS, Abuodeh YA, Ahmed KA, Strom T, Frakes JM, Trotti A, Harrison LB, Caudell JJ. Medicaid Independently Predicts for Poor Outcome in Head and Neck Malignancies. Int J Rad Oncol Biol Phys. 2015; 93(3):E370.	Abstract.
**80.**	Naghavi AO, Echevarria MI, Grass GD, Strom TJ, Abuodeh YA, Ahmed KA, Kim Y, Trotti AM, Harrison LB, Yamoah K, Caudell JJ. Having Medicaid insurance negatively impacts outcomes in patients with head and neck malignancies. Cancer. 2016; 122(22):3529-3537.	Head and neck patient delay without separating oral cancer in the analysis.
**81.**	Naghavi AO, Echevarria M, Strom T, Abuodeh YA, Ahmed KA, Venkat PS, Trotti A, Harrison LB, Green BL, Yamoah K, Caudell JJ. Race Is Associated With Poor Outcomes in Head and Neck Cancer. Int J Rad Oncol Biol Phys. 2016; 96(2): E407.	Abstract.

**82.**	Naghavi AO, Echevarria MI, Strom TJ, Abuodeh YA, Ahmed KA, Venkat PS, Trotti A, Harrison LB, Green BL, Yamoah K, Caudell JJ. Treatment delays, race, and outcomes in head and neck cancer. Cancer Epidemiol. 2016; 45:18-25.	Head and neck patient delay without separating oral cancer in the analysis.
**83.**	Nash R, Hughes J, Sandison A, Stewart S, Clarke P, Mace A. Factors associated with delays in head and neck cancer treatment: case-control study. J Laryngol Otol. 2015; 129(4):383-5.	Professional delay.
**84.**	Nieminen M, Atula T, Bäck L, Mäkitie A, Jouhi L, Aro K. Factors influencing patient and health care delays in Oropharyngeal Cancer. J Otolaryngol — Head Neck Sur. 2020; 49(1).	The study is about time intervals from diagnosis to treatment. It does not report the socioeconomic factors of the sample.
**85.**	Ning MS, Gomez DR, Shah AK, Kim CR, Palmer MB, Thaker NG, Grosshans DR, Liao Z, Chapman BV, Brooks ED, Tang C, Rosenthal DI, Garden AS, Frank SJ, Gunn GB. The Insurance Approval Process for Proton Radiation Therapy: A Significant Barrier to Patient Care. Int J Radiat Oncol Biol Phys. 2019; 104(4):724-733.	The study is about the time for insurance authorization to treatment of cancer.
**86.**	Nocon CC, Ajmani GS, Bhayani MK. A contemporary analysis of racial disparities in recommended and received treatment for head and neck cancer. Cancer. 2020; 126(2):381-389.	The study is not about time-to-treatment but only about socioeconomic factors.
**87.**	Nocon CC, Liederbach E, Sisco M, Yao K, Gerber ME, Lewis CM, Bhayani MK. Factors Affecting Delays to Surgery for Oral Tongue Carcinoma in the National Cancer Data Base. Otolaryngol Head Neck Surg. 2014; 151(1_suppl):P56.	Abstract.
**88.**	Overgaard J, Jovanovic A, Godballe C, Grau Eriksen J. The Danish Head and Neck Cancer database. Clin Epidemiol. 2016; 8:491-496.	The study is about tumor characteristics and treatment of head and neck cancer.
**89.**	Pakravan F, Abbasi F, Garshasbi MA, Isfahani MN. Relationship between oral cancer stage and elapsed time from the onset of signs and symptoms to diagnosis and treatment. Cancer Treat Res Commun. 2021; 28:100428.	Professional and patient delay (interval of first symptoms and diagnosis).
**90.**	Pandrowala S, Ramraj D, Shankar R, Chopra S, Das A, Mishra A, Pandey D. Impact of preoperative COVID infection on the outcomes of planned curative-intent cancer surgeries in the second wave of the pandemic from a tertiary care center in India. J Surg Oncol. 2022; 125(2):107-112.	The study is about treatment delays due to infection by COVID-19 patients with different types of cancer.
**91.**	Patel PA, Christopher E, Fundakowski MD. Impact of facility and patient demographic on time to treatment initiation in head and neck cancer. Otolaryngol Head Neck Surg. 2018; 159(1_suppl):P206–P250.	Abstract.
**92.**	Patel UA, Brennan TE. Disparities in head and neck cancer: assessing delay in treatment initiation. Laryngoscope. 2012; 122(8):1756-60.	Head and neck patient delay without separating oral cancer in the analysis.
**93.**	Patil RD, Meinzen-Derr JK, Hendricks BL, Patil YJ. Improving access and timeliness of care for veterans with head and neck squamous cell carcinoma: A multidisciplinary team's approach. Laryngoscope. 2016; 126(3):627-31.	The study compares two groups before and after multidisciplinary care intervention on the improvement of time-to-treatment in head and neck cancers.
**94.**	Patni N, Hota A, Patni A, Misra P. Cancer during Corona Pandemic: Plight of cancer patients when two evils join hands. Curr Probl Cancer. 2021; 45(3):100671.	The study is about different types of cancer.
**95.**	Peacock ZS, Pogrel MA, Schmidt BL. Exploring the reasons for delay in treatment of oral cancer. J Am Dent Assoc. 2008; 139(10):1346-52.	The study describes time intervals from diagnosis to treatment. It does not report the socioeconomic factors of the sample.
**96.**	Pepin A, Goyal S, Thakkar P, Siegel R, Joshi A, Goodman J, Ojong-Ntui M, Rao YJ. Use of Distress Thermometer to Predict Utilization of Emergency Room Services and Treatment Delays in Head and Neck Cancer Patients Undergoing Radiation Therapy. Int J Rad Oncol Biol Phys. 2020; 108(3):E221.	Abstract.
**97.**	Perlow H, Ramey S, Jaffe A, Silver B, Kwon D, Chinea F, Samuels S, Yechieli R. Examining head and neck cancer treatment delay in an ethnically diverse population: A look at the impact of hispanic ethnicity, spanish language preference, and treatment setting. J Radiat Oncol. 2017; 6(3):225-245.	Abstract.
**98.**	Perlow HK, Ramey SJ, Silver B, Kwon D, Chinea FM, Samuels SE, Samuels MA, Elsayyad N, Yechieli R. Assessment of Oropharyngeal and Laryngeal Cancer Treatment Delay in a Private and Safety Net Hospital System.	The study is not about squamous cell carcinoma.

	Otolaryngol Head Neck Surg. 2018; 159(3):484-493.	
**99.**	Perni S, Busse PM, Chan AW, Connolly RJ, Shih H. A. Time to Insurance Approval and Treatment for Proton Beam Therapy for Head and Neck Cancers. Int J Rad Oncol Biol Phys. 2020; 106(5):1208–1209.	Abstract.
**100.**	Peters ES, Brashear MM, Ferguson TF. Treatment and survival after head and neck cancer diagnosis by race in Louisiana's public hospital system. *Cancer* Epidemiol Biomarkers Prev 1. 2015; 24 (10_Supplement): A93.	Abstract.
**101.**	Pham A, Lai P, Phuong C, Batth S, Chan C, Schechter N, Trakul N, Garsa AA, Jennelle R. Definitive Radiation Treatment Compliance and Outcomes at a Safety Net Hospital. Int J Rad Oncol Biol Phys. 2019; 105(1):E449.	Abstract.
**102.**	Polesel J, Furlan C, Birri S, Giacomarra V, Vaccher E, Grando G, Gobitti C, Navarria F, Schioppa O, Minatel E, Bidoli E, Barzan L, Franchin G. The impact of time to treatment initiation on survival from head and neck cancer in north-eastern Italy. Oral Oncol. 2017; 67:175-182.	Head and neck patient delay without separating oral cancer in the analysis.
**103.**	Raman A, Sen N, Ritz E, Fidler MJ, Revenaugh P, Stenson K, Al-Khudari S. Heterogeneity in the clinical presentation, diagnosis, and treatment initiation of p16-positive oropharyngeal cancer. Am J Otolaryngol. 2019; 40(5):626-630.	The study is not about time-to-treatment but only about socioeconomic factors.
**104.**	Raman S, Shafie AA, Abraham MT, Kiong SC, Maling TH, Rajendran S, Cheong SC. Time-to-Treatment of Oral Cancer and Potentially Malignant Oral Disorders: Findings in Malaysian Public Healthcare. Dent J (Basel). 2022 Oct 24;10(11):199.	The study is about time intervals from diagnosis to treatment. It does not report socioeconomic factors of the sample.
**105.**	Richardson PA, Kansara S, Chen GG, Sabichi A, Sikora AG, Parke RB, Donovan DT, Chiao E, Sandulache VC. Treatment Patterns in Veterans with Laryngeal and Oropharyngeal Cancer and Impact on Survival. Laryngoscope Investig Otolaryngol. 2018; 3(4):275-282.	The study is about laryngeal and oropharyngeal cancers. It does not separate oral cancer in the analysis.
**106.**	Rick T, Habtamu B, Tigeneh W, Abreha A, van Norden Y, Grover S, Assefa M, Incrocci L. Patterns of Care of Cancers and Radiotherapy in Ethiopia. J Glob Oncol. 2019; 5:1-8.	The study is about overall types of cancer, including head and neck cancer.
**107.**	Rosskamp M, Verbeeck J, Sass V, Gadeyne S, Verdoodt F, De Schutter H. Social Inequalities in Cancer Survival in Belgium: A Population-Based Cohort Study. Cancer Epidemiol Biomarkers Prev. 2021; 30(1):45-52.	The study is about overall types of cancer, including head and neck cancer.
**108.**	Rotsides JM, Oliver JR, Moses LE, Tam M, Li Z, Schreiber D, Jacobson AS, Hu KS, Givi B. Socioeconomic and Racial Disparities and Survival of Human Papillomavirus-Associated Oropharyngeal Squamous Cell Carcinoma. Otolaryngol Head Neck Surg. 2021; 164(1):131-138.	The study is not about time-to-treatment but only about socioeconomic factors.
**109.**	Rygalski CJ, Zhao S, Eskander A, Zhan KY, Mroz EA, Brock G, Silverman DA, Blakaj D, Bonomi MR, Carrau RL, Old MO, Rocco JW, Seim NB, Puram SV, Kang SY. Time to Surgery and Survival in Head and Neck Cancer. Ann Surg Oncol. 2021; 28(2):877-885.	Head and neck patient delay without separating oral cancer in the analysis.
**110.**	Sargeran K. Treatment delay among oral cancer patients in Tehran, Iran. J Craniomaxillofac Res. 2015; 1(3-4):37-41.	The study is aboutthe time intervals from diagnosis to treatment. It does not report socioeconomic factors of the sample.
**111.**	Sargeran K, Murtomaa H, Safavi SMR. The role of socioeconomic factors and dental service utilization in treatment delay among oral cancer patients in Tehran, Iran. Oral Oncology. 2013; 49:S107.	Abstract.
**112.**	Sharp L, Lewin F, Hellborg H, Lundgren J, Hemmingsson E, Rutqvist LE. When does my treatment start?—The continuum of care for patients with head and neck cancer. Radiother Oncol. 2002; 63(3):293-7.	Professional delay (diagnosis delay).
**113.**	Schoonbeek RC, Bult FFS, Plaat BEC, Witjes MJH, van der Hoorn A, van Dijk BAC, Halmos GB. Incidental findings during the diagnostic work-up in the head and neck cancer pathway: Effects on treatment delay and survival. Oral Oncol. 2021; 118:105350.	Head and neck patient delay without separating oral cancer in the analysis.
**114.**	Schoonbeek RC, de Vries J, Bras L, Sidorenkov G, Plaat BEC, Witjes MJH, van der Laan BFAM, van den Hoek JGM, van Dijk BAC, Langendijk JA, Halmos GB. The effect of treatment delay on quality of life and overall survival in head and neck cancer patients. Eur J Cancer Care (Engl). 2022; 31(4):e13589.	Head and neck patient delay without separating oral cancer in the analysis.
**115.**	Schoonbeek RC, Festen S, Rashid R, van Dijk BAC, Halmos GB, van der Velden LA. Impact of Delay on Hospitalization in Older Patients With Head and	Head and neck patient delay without separating oral cancer in the analysis.

	Neck Cancer: A Multicenter Study. Otolaryngol Head Neck Surg. 2022; 167(4):678-687.	
**116.**	Schoonbeek RC, de Jel DVC, van Dijk BAC, Willems SM, Bloemena E, Hoebers FJP, van Meerten E, Verbist BM, Smeele LE, Halmos GB, Merkx MAW, Siesling S, De Bree R, Takes RP; Dutch Head, Neck Society, the COVID, Cancer-NL consortium. Fewer head and neck cancer diagnoses and faster treatment initiation during COVID-19 in 2020: A nationwide population-based analysis. Radiother Oncol. 2022; 167:42-48.	Head and neck patient delay without separating oral cancer in the analysis.
**117.**	Schutte HW, van den Broek GB, Steens SCA, Hermens RPMG, Honings J, Marres HAM, Merkx MAW, Weijs WLJ, Arens AIJ, van Engen-van Grunsven ACH, van Herpen CML, Kaanders JHAM, van den Hoogen FJA, Takes RP. Impact of optimizing diagnostic workup and reducing the time to treatment in head and neck cancer. Cancer. 2020; 126(17):3982-3990.	Head and neck patient delay without separating oral cancer in the analysis.
**118.**	Sethi HK, Weinsheim T, Brennan M, Walker E, Fundakowski CE. Optimizing head and neck cancer treatment in the community setting. Head Neck Surg. 2021; 165(1).	Abstract.
**119.**	Sharma A, Madan R, Bharat B, Behera M, Mohanti BK, Rath GK. Multi-modality cancer care- what is planned & what is delivered. Radiot Oncol. 2012; 103: S375.	Abstract.
**120.**	Sharma S, Bekelman JE, Lin A, Roman B, Mitra N, Swisher-McClure SD. Risk Factors for Prolonged Diagnosis to Treatment Interval (DTI) Among Patients With Head and Neck Squamous Cell Carcinoma (HNSCC). Int J Rad Oncol Biol Phys. 2014; 90(1):S558.	Abstract.
**121.**	Shin JY, Yoon JK, Shin AK, Diaz AZ. The influence of insurance status on treatment and outcomes in oral cavity cancer: an analysis on 46,373 patients. Int J Oral Maxillofac Surg. 2018; 47(10):1250-1257.	The study is not about time-to-treatment but only about socioeconomic factors.
**122.**	Simons PA, Ramaekers B, Hoebers F, Kross KW, Marneffe W, Pijls-Johannesma M, Vandijck D. Cost-Effectiveness of Reduced Waiting Time for Head and Neck Cancer Patients due to a Lean Process Redesign. Value Health. 2015; 18(5):587-96.	Head and neck patient delay without separating oral cancer in the analysis.
**123.**	Sineshaw HM, Ellis MA, Yabroff KR, Han X, Jemal A, Day TA, Graboyes EM. Association of Medicaid Expansion Under the Affordable Care Act With Stage at Diagnosis and Time to Treatment Initiation for Patients With Head and Neck Squamous Cell Carcinoma. JAMA Otolaryngol Head Neck Surg. 2020; 146(3):247-255.	Head and neck patient delay without separating oral cancer in the analysis.
**124.**	Souza Cruz AC, Franzolin SOB, Pereira AAC, Hanneman JAC, Beijo LA, Cruz JRS. Oral squamous cell carcinoma: survival, recurrence and death. Acta Scientiarum. 2014; 36(2): 273-279.	The study compares time to treatment with death from oral cancer.
**125.**	Subramanian S, Chen A. Treatment patterns and survival among low-income medicaid patients with head and neck cancer. JAMA Otolaryngol Head Neck Surg. 2013;139(5):489-95.	Head and neck patient delay without separating oral cancer in the analysis.
**126.**	Suzuki I, Cullen KJ, Mehra R, Bentzen S, Goloubeva OG. 2019. Racial disparities in outcome among head and neck cancer patients in the United States: An analysis using SEER-Medicare linked database. J Clin Oncol. 37(15):6051	Abstract.
**127.**	Sawaf T, Virgen CG, Renslo B, Farrokhian N, Yu KM, Somani SN, et al. Association of social-ecological factors with delay in time to initiation of postoperative radiation therapy: a prospective cohort study. JAMA Otolaryngol Head Neck Surg. 2023;149(6):477–84.	Head and neck patient delay without separating oral cancer in the analysis.
**128.**	Szewczyk M, Pazdrowski J, Golusiński P, Pazdrowski P, Więckowska B, Golusiński W. The impact of the COVID-19 pandemic on the management of head and neck cancer patients at a tertiary care institution in Poland. Contemp Oncol (Pozn). 2021; 25(4):264-269.	Head and neck cancer diagnoses and treatment initiation during COVID-19.
**129.**	Tan JY, Otty ZA, Vangaveti VN, Buttner P, Varma SC, Joshi AJ, Kelly J, Collins M, Sabesan SS. A prospective comparison of times to presentation and treatment of regional and remote head and neck patients in North Queensland, Australia. Intern Med J. 2016; 46(8):917-24.	Head and neck patient delay without separating oral cancer in the analysis.
**130.**	Thaduri A, Dungala D, Panuganti A, Majumdar KS, Sehrawat A. Addressing the challenges of head and neck cancer care in India - A compelling need of the hour. Oral Oncol. 2022; 124:105638.	Opinion Article.
**131.**	Tham T, Leung E, Khaymovich J, Mitha S, Olson C, Wotman M, Teegala S,	Head and neck patient delay without

	Coury J, Costantino P. Evaluation of Time to Surgery and Its Effect on Prognosis in Head and Neck Cancer. Oncol Res Treat 2019; 42:387-395.	separating oral cancer in the analysis.
**132.**	Thompson JA, Joy R, Lubek JE, Mehra R, Molitoris JK, Taylor RJ, Wolf JS, Witek ME, Hatten KM. Evaluating the impact of the coronavirus (COVID-19) pandemic on treatment paradigms in head and neck cancer at a tertiary care hospital. Clin Cancer Res. 2020; 26 (18_Supplement): PO–017.	Abstract.
**133.**	Tirelli G, Boscolo-Rizzo P, Pelloso L, Gardenal N, Giudici F, Marcuzzo AV, Tofanelli M. Impact of time-to-surgery on survival and quality of life in oral cancer. Am J Otolaryngol. 2023 Nov-Dec;44(6):103984.	The study not report the socioeconomic factors of the sample.
**134.**	Toustrup K, Lambertsen K, Birke-Sørensen H, Ulhøi B, Sørensen L, Grau C. Reduction in waiting time for diagnosis and treatment of head and neck cancer - a fast track study. Acta Oncol. 2011; 50(5):636-41.	Head and neck patient delay without separating oral cancer in the analysis.
**135.**	Tumati V, Hoang L, Sumer BD, Truelson JM, Myers LL, Khan S, Hughes RS, Nedzi L, Sher DJ. Association between treatment delays and oncologic outcome in patients treated with surgery and radiotherapy for head and neck cancer. Head Neck. 2019; 41(2):315-321.	Head and neck patient delay without separating oral cancer in the analysis.
**136.**	Vaddepally RK, Damarla V, Singal B, Winn J, Winegarden JD, Narayan S, Akervall JA. Impact of single day multidisciplinary clinics on the lead time from diagnosis to initiation of treatment in head and neck cancers. J Clin Oncol. 2015; 33:15: e17523.	Abstract.
**137.**	van Harten MC, De Haan RR, Hoebers FJP, Van Dijk BAC, Aaronson N, Van Den Brekel MWM. Treatment waiting times for head and neck cancer in the netherlands: Their relation to survival and stress. 5th World Congress of the International Academy of Oral Oncology (IAOO). Head Neck. 2015; 37 (Suppl 1):E1-E227.	Abstract.
**138.**	van Harten MC, Hoebers FJ, Kross KW, van Werkhoven ED, van den Brekel MW, van Dijk BA. Determinants of treatment waiting times for head and neck cancer in the Netherlands and their relation to survival. Oral Oncol. 2015; 51(3):272-8.	Head and neck patient delay without separating oral cancer in the analysis.
**139.**	van Harten MC, de Ridder M, Hamming-Vrieze O, Smeele LE, Balm AJ, van den Brekel MW. The association of treatment delay and prognosis in head and neck squamous cell carcinoma (HNSCC) patients in a Dutch comprehensive cancer center. Oral Oncol. 2014; 50(4):282-90.	Head and neck patient delay without separating oral cancer in the analysis.
**140.**	Venchiarutti, R.; Clark, J. R.; Palme, C. E.; Young, J. M. Patient health literacy correlates with times to diagnosis of head and neck cancer. Asia Pac J Clin Oncol. 2021; 17(S5): 42.	Abstract.
**141.**	Wadia RJ, Yao X, Deng Y, Li J, Maron S, Connery D, Gunduz-Bruce H, Rose MG. The effect of pre-existing mental health comorbidities on the stage at diagnosis and timeliness of care of solid tumor malignances in a Veterans Affairs (VA) medical center. Cancer Med. 2015; 4(9):1365-73.	The study is about overall types of cancer, including head and neck cancer.
**142.**	Wang CP, Liao LJ, Chiang CJ, Hsu WL, Kang CJ, Wang CC, Chen PR, Chen TC, Huang WW, Chien CY. Patients with oral cancer do not undergo surgery as primary treatment: A population-based study in Taiwan. J Formos Med Assoc. 2020; 119(1 Pt 3):392-398.	The study describes time intervals from diagnosis to treatment. It does not report the socioeconomic factors of the sample.
**143.**	Webb CJ, Benton J, Tandon S, Jones TM, Roland NJ. Head and neck cancer waiting times. Clin Otolaryngol. 2007; 32(4):293-6.	Head and neck patient delay without separating oral cancer in the analysis.
**144.**	Waaijer A, Terhaard CHJ, Dehnad H, Hordijk G-J, van Leeuwen MS, Raaymakers CPJ, Lagendijk JJW. Waiting times for radiotherapy: consequences of volume increase for the TCP in oropharyngeal carcinoma. Radiot Oncol. 2003; 66(3), 271–276.	The study is about time intervals from diagnosis to treatment. It does not report the socioeconomic factors of the sample.
**145.**	Worrall SF, Corrigan M. An audit of one surgeon's experience of oral squamous cell carcinoma using computerised malignancy database. Ann R Coll Surg Engl. 1995; 77(5):332-6.	The study is about time intervals from diagnosis to treatment. It does not report the socioeconomic factors of the sample.
**146.**	Xiao R, Ward MC, Yang K, Adelstein DJ, Koyfman SA, Prendes BL, Burkey BB. Increased pathologic upstaging with rising time to treatment initiation for head and neck cancer: A mechanism for increased mortality. Cancer. 2018; 124(7):1400-1414.	Head and neck patient delay without separating oral cancer in the analysis.
**147.**	Yarn C, Schwartz DL. Treatment Compliance and Insurance Status in Head and Neck Radiation Therapy Patients. Int J Rad Oncol Biol Phys. 2018;	Abstract.

	100(5):P1341.	
**148.**	Zavarez LB, Stramandinoli-Zanicotti RT, Sassi LM, Ramos GH, Schussel JL, Torres-Pereira CC. The interval since first symptoms until diagnosis of squamous cell carcinoma in the head and neck region is still a problem in southern Brazil. Med Oral Patol Oral Cir Bucal. 2020; 25(6):e769-e774.	Patient delay (interval from first symptoms until diagnosis).
**149.**	Žumer B, Pohar Perme M, Jereb S, Strojan P. Impact of delays in radiotherapy of head and neck cancer on outcome. Radiat Oncol. . 2020; 15(1):202.	Head and neck patient delay without separating oral cancer in the analysis.

Box S3. Descriptive table of socioeconomic variables found in the included articles, divided by the way the outcome treatment delay was presented (categorical or continuous).

Study	Definition of treatment delay (sample)	Sex by group	Age by group	Outcome by category	Available data (ES; 95%CI)	p-value
			**INSURANCE**			
			**Categorical**			
Sharma et al., 2016 [23]	**Group 1:** DTI< 30 days (n=3,020) **Group 2:** DTI ≥ 30 days (n=3,586)	**Group 1:** Male: 2,482 (82.2%) Female: 538 (17.8%) **Group 2:** Male: 2,951 (82.3%) Female: 635 (17.7%)	**Group 1:** <65: 2,319 (76.8%) ≥65: 701 (23.2%) **Group 2:** <65: 2,736 (76.3%) ≥65: 850 (23.7%)	**Group 1:** Commercial insurance: 1,737 (57.5%) Medicare: 720 (23.8%) Medicaid: 264 (8.7%) Government: 58 (1.9%) Uninsured: 172 (5.7%) Unknown: 69 (2.3%) **Group 2:** Commercial insurance: 1,772 (49.4%) Medicare: 882 (24.6%) Medicaid: 437 (12.2%) Government: 86 (2.4%) Uninsured: 323 (9.0%) Unknown: 86 (2.4%)	Commercial insurance: Ref. Medicare (OR: 1.27; 1.07 to 1.51) Medicaid (OR:1.58; 1.32 to1.88) Uninsured (OR: 1.90; 1.55 to 2.33) Government (OR:1.38; 0.97 to 1.95) Unknown (OR: 1.25; 0.90 to 1.75)	Commercial insurance: Ref.[a] Medicare**: p< 0.05** Medicaid: **p< 0.001** Uninsured: **p< 0.001** Government: p> 0.05 Unknown: p> 0.05
Morse et al. 2018 [24]	**Group 1: No delay** (First and second quartiles for each interval of days; n=2,012) **Group 2: Delay** (Fourth quartile for each interval of days; n=1,032).	**Group 1:** Male: 1,701 (85%) Female: 311 (15%) **Group 2:** Male: 829 (80%) Female: 203 (20%)	**Group 1:** ≤60 years:1,253 (62%) >60 years: 759 (38%) **Group 2:** ≤60 years: 634 (61%) >60 years: 398 (39%)	**Group 1:** Not insured: 115 (6%) Private insurance: 1,187 (59%) Medicaid: 156 (8%) Medicare: 527 (26%) Insurance unknown: 27(1%) **Group 2:** Not insured: 120 (12%) Private insurance: 456 (44%) Medicaid: 138 (13%) Medicare: 304 (29%) Unknown: 14 (1%)	Private insurance: Ref. Not insured (OR: 2.71;2.00 to 3.68) Medicaid (OR: 1.97; 1.49 to 2.60) Medicare (OR: 1.39; 1.11 to 1.73) Unknown (OR:1.30; 0.65 to 2.59)	Private insurance: Ref.[a] Not insured: **p< 0.001** Medicaid: **p< 0.001** Medicare: **p= 0.004** Unknown: p=0.458
			**Continuous**			
Fujiwara et al., 2017 [12]	The fourth quartile of each treatment interval was used as an indicator of delayed treatment. **Median=** 30 days.	NR**	NR**	Not insured: 237 (4.9%) Private insurance: 2,114 (43.4%) Medicaid: 360 (7.4%) Medicare: 2,000 (41.1%) Other/unknown: 157 (3.2%)	Other/unknown: Ref. Not insurance (OR: 2.24; 1.33 to 3.77) Private Insurance (OR: 1.21; 0.79 to 1.85) Medicaid (OR: 2.52; 1.55 to 4.08) Medicare (OR: 1.27; 0.82 to 1.95)	Other/unknown: Ref.[a] Not insurance: **p=0.002** Private Insurance: p=0.39 Medicaid: **p<0.001** Medicare: p=0.28
Morse et al. 2018	Patients were classified as	NR**	NR**	Not insured: 136 (4%)	Private insurance: Ref.	Private insurance: Ref.[a]

[25]	delayed (fourth quartile) vs not delayed (first and second quartiles) for each interval of time to treatment.<br><br>**Median=** 27 days.			Private insurance: 2,484 (67%)<br>Medicaid: 216 (6%)<br>Medicare: 827 (22%)<br>Insurance status Unknown: 45 (1%)	Not insured (OR:1.19; 0.68 to 2.09)<br>Medicaid (OR: 2.49; 1.55 to 3.99)<br>Medicare (OR: 1.28; 0.94 to 1.75)<br>Unknown (OR: 0.96; 0.36 to 2.58)	Not insured: p=0.543<br>Medicaid: **p< 0.001**<br>Medicare: p= 0.112<br>Unknown: p=0.0943
Goel et al., 2019 [13]	Time to treatment was presented as a continuous variable (number of days).<br><br>**Median=** 26 days.	NR**	NR**	Medicare: 756 (21.3%)<br>Medicaid: 207 (5.83%)<br>Private/managed care/other government: 2,455 (69.2%)<br>None: 98 (2.8%)<br>Unknown: 34 (1.0%)	Private/managed care/other government: Ref.<br>Medicare: mean days (Coefficient**:** 1.14; −0.98 to 3.26)<br>Medicaid: mean days (Coefficient**:** 3.12; 0.10 to 6.14)<br>None: mean days (Coefficient**:** 4.95; 0.71 to 9.20)<br>Unknown: mean days (Coefficient**:** −2.76; −9.80 to 4.28)	Private/managed care/other government: Ref.[a]<br>Medicare: p=0.29<br>Medicaid: **p=0.04**<br>None: **p=0.02**<br>Unknown: p=0.44
			**FACILITY TYPE**			
			**Categorical**			
Sharma et al., 2016 [23]	**Group 1:** DTI< 30 days (n=3,020)<br><br>**Group 2:** DTI ≥ 30 days (n=3,586)	**Group 1:**<br>Male: 2,482 (82.2%)<br>Female: 538 (17.8%)<br><br>**Group 2:**<br>Male: 2,951 (82.3%)<br>Female: 635 (17.7%)	**Group 1:**<br><65: 2,319 (76.8%)<br>≥65: 701 (23.2%)<br><br>**Group 2:**<br><65: 2,736 (76.3%)<br>≥65: 850 (23.7%)	**Group 1:**<br>Academic: 1,111 (36.8%)<br>Community: 1,909 (63.2%)<br><br>**Group 2:**<br>Academic: 1,632 (45.5%)<br>Community: 1,954 (54.5%)	Community: Ref.<br>Academic (OR: 1.26; 1.13 to 1.42)	Community: Ref.[a]<br>Academic= **p<0.001**
Morse et al., 2018 [24]	**Group 1: No delay** (First and second quartiles for each interval of days; n=2,012)<br><br>**Group 2: Delay** (Fourth quartile for each interval of days; n=1,032).	**Group 1:**<br>Male: 1,701 (85%)<br>Female: 311 (15%)<br><br>**Group 2:**<br>Male: 829 (80%)<br>Female: 203 (20%)	**Group 1:**<br>≤60 years:1,253 (62%)<br>>60 years: 759 (38%)<br><br>**Group 2:**<br>≤60 years: 634 (61%)<br>>60 years: 398 (39%)	**Group 1:**<br>Community cancer program: 159 (8%)<br>Comprehensive community cancer program: 751 (37%)<br>Academic/research program: 833 (41%)<br>Integrated network cancer program: 269 (13%)<br><br>**Group 2:**<br>Community cancer program: 78 (8%)<br>Comprehensive community cancer program: 297 (29%)<br>Academic/research program: 525 (51%)<br>Integrated network cancer program: 132 (13%)	Community cancer program: Ref.<br>Academic/research program (OR: 1.52; 1.03 to 2.26)<br>Comprehensive community cancer program (OR: 0.98; 0.68 to 1.42)<br>Integrated network cancer program (OR: 1.33; 0.85 to 2.08)	Community cancer program: Ref.[a]<br>Academic/research program: **p=0.037**<br>Comprehensive community cancer program: p=0.933<br>Integrated network cancer program: p=0.213
			**Continuous**			
Fujiwara et al.,	The fourth quartile of each	NR**	NR**	Community cancer program: 269 (5.5%)	Community cancer program: Ref. *	Community cancer program: Ref.[a]

2017 [12]	treatment interval was used as an indicator of delayed treatment.<br><br>**Median=** 30 days.			Comprehensive community cancer program: 1,819 (37.4%)<br>Academic/research program: 2,718 (55.8)<br>Other: 62 (1.3%)	Comprehensive community cancer program (OR: 1.16; 0.79 to 1.70)<br>Academic/research program (OR: 2.17; 1.49 to 3.15)<br>Other (OR: 1.71; 0.82 to 3.58)	Comprehensive community cancer program: p=0.44<br>Academic/research program: **p<0.001**<br>Other: p=0.15
Morse et al., 2018 [25]	Patients were classified as delayed (fourth quartile) vs not delayed (first and second quartiles) for each interval of time to treatment.<br><br>**Median=** 27 days.	NR**	NR**	Community cancer program: 252 (7%)<br>Comprehensive community cancer program: 1,101 (30%)<br>Academic/research program: 2,006 (54%)<br>Integrated network cancer program: 349 (9%)	Community cancer program: Ref.<br>Comprehensive community cancer program (OR: 1.24; 0.65 to 2.35)<br>Academic/research program (OR: 1.79; 0.92 to 3.51)<br>Integrated network cancer program (OR: 1.38; 0.65 to 2.93)	Community cancer program: Ref.[a]<br>Comprehensive community cancer program: p= 0.515<br>Academic/research program: p=0.088<br>Integrated network cancer program: p= 0.408
Goel et al., 2019 [13]	Time to treatment was presented as a continuous variable (number of days).<br><br>**Median=** 26 days	NR**	NR**	Academic: 2,015 (56.8%)<br>Comprehensive community: 965 (27.2%)<br>Community: 177 (5.0%)<br>Other: 393 (11.1%)	Academic: Ref.<br>Comprehensive community (Coefficient: −4.95; −6.65 to −3.25)<br>Community (Coefficient: −5.28; −8.57 to −1.99)<br>Other (Coefficient: −1.15; −3.69 to 1.38)	Academic: Ref.[a]<br>Comprehensive community: **p<0.001**<br>Community: **p= 0.002**<br>Other: p=0.37
**HOSPITAL SERVICES VOLUME**						
**Categorical**						
Sharma et al., 2016 [23]	**Group 1:** DTI< 30 days (n=3,020)<br><br>**Group 2:** DTI ≥ 30 days (n=3,586)	**Group 1:** Male: 2,482 (82.2%) Female: 538 (17.8%)<br><br>**Group 2:** Male: 2,951 (82.3%) Female: 635 (17.7%)	**Group 1:** <65: 2,319 (76.8%) ≥65: 701 (23.2%)<br><br>**Group 2:** <65: 2,736 (76.3%) ≥65: 850 (23.7%)	**Group 1:** Low case-volume: 2,365 (78.3%) High case-volume: 655 (21.7%)<br><br>**Group 2:** Low case-volume: 2,539 (70.8%) High case-volume: 1,047 (29.2%)	Low case-volume: Ref.<br>High case-volume (OR: 1.38; 1.21 to 1.58)	Low case-volume: Ref.[a]<br>High case-volume: **p<0.001**
Morse et al., 2018 [24]	**Group 1: No delay** (First and second quartiles for each interval of days; n=2,012)<br><br>**Group 2: Delay** (Fourth quartile for each interval of days; n=1,032).	**Group 1:** Male: 1,701 (85%) Female: 311 (15%)<br><br>**Group 2:** Male: 829 (80%) Female: 203 (20%)	**Group 1:** ≤60 years:1,253 (62%) >60 years: 759 (38%)<br><br>**Group 2:** ≤60 years: 634 (61%) >60 years: 398 (39%)	**Group 1:** Low: 345 (17%) Medium: 1,123 (56%) High: 544 (27%)<br><br>**Group 2:** Low: 167 (16%) Medium: 529 (51%) High: 336 (33%)	Low: Ref.<br>Medium (OR: 0.81; 0.62 to 1.07)<br>High (OR: 0.93; 0.67 to 1.29)	Low: Ref.[a]<br>Medium: p=0.139<br>High: p=0.654
Tsai et al., 2017 [11]	**Group 1:** <=30 days (n = 18,193)<br><br>**Group 2:** 31 to 120 days (n = 2,498)	**Group 1:** Male: 16,704 (85.6%) Female: 1,489 (84.7%)<br><br>**Group 2:**	**Group 1:** <=44: 4,304 (86.1%) 45-54: 6,337 (85.9%) 55-64: 4,296 (86.1%)	**Group 1:** Low:4,665 (85.2%) Middle: 8,901 (85.2%) High: 4,627 (86.7%)<br><br>**Group 2:**	NR**	**p<0.001**<br><br>χ2 test

	**Group 3:** >120 days<br>(n = 572)	Male: 2,296 (11.8%)<br>Female: 202 (11.5%)<br><br>**Group 3:**<br>Male: 505 (2.6%)<br>Female: 67 (3.8%)	≥65: 3,257 (83.6%)<br><br>**Group 2:**<br><=44:567 (11.3%)<br>45-54: 842 (11.4%)<br>55-64: 554 (11.1%)<br>≥65: 535 (13.7%)<br><br>**Group 3:**<br><=44: 131 (2.6%)<br>45-54: 197 (2.7%)<br>55-64:138 (2.8%)<br>≥65: 106 (2.7%)	Low: 603 (11.0%)<br>Middle: 1,286 (12.3%)<br>High: 609 (11.4%)<br><br>**Group 3:**<br>Low: 206 (3.8%)<br>Middle: 263 (2.5%)<br>High: 103 (1.9%)		
			**Continuous**			
Morse et al. 2018 [25]	Patients were classified as delayed (fourth quartile) vs not delayed (first and second quartiles) for each interval of time to treatment.<br><br>**Median=** 27 days.	NR**	NR**	Low: 560 (15%)<br>Medium: 1,832 (49%)<br>High: 1,316 (35%)	Low: Ref.<br>Medium (OR: 1.09; 0.68 to 1.76)<br>High (OR: 1.09; 0.65 to 1.83)	Low: Ref.[a]<br>Medium: p=0.714<br>High: p=0.753
			**HOSPITAL LEVEL**			
			**Categorical**			
Tsai et al., 2017 [11]	**Group 1:** <=30 days<br>(n = 18,193)<br><br>**Group 2:** 31 to 120 days<br>(n = 2,498)<br><br>**Group 3:** >120 days<br>(n = 572)	**Group 1:**<br>Male: 16,704 (85.6%)<br>Female: 1,489 (84.7%)<br><br>**Group 2:**<br>Male: 2,296 (11.8%)<br>Female: 202 (11.5%)<br><br>**Group 3:**<br>Male: 505 (2.6%)<br>Female: 67 (3.8%)	**Group 1:**<br><=44: 4,304 (86.1%)<br>45-54: 6,337 (85.9%)<br>55-64: 4,296 (86.1%)<br>≥65: 3,257 (83.6%)<br><br>**Group 2:**<br><=44:567 (11.3%)<br>45-54: 842 (11.4%)<br>55-64: 554 (11.1%)<br>≥65: 535 (13.7%)<br><br>**Group 3:**<br><=44: 131 (2.6%)<br>45-54: 197 (2.7%)<br>55-64:138 (2.8%)<br>≥65: 106	**Group 1:**<br>Medical centers: 14,037 (85.9%)<br>Regional hospitals: 4,065 (84.4%)<br>Others: 91 (81.3%)<br><br>**Group 2:**<br>Medical centers: 1,896 (11.6%)<br>Regional hospitals: 589 (12.2%)<br>Others: 13 (11.6%)<br><br>**Group 3:**<br>Medical centers: 403 (2.5%)<br>Regional hospitals: 161 (3.4%)<br>Others: 8 (7.1%)	NR**	**p<0.001**<br><br>χ2 test

			(2.7%)			
Su et al., 2021 [26]	**Group 1:** <=3 weeks (n= 3,267)<br><br>**Group 2:** >3 to 6 weeks (n= 1,700)<br><br>**Group 3:** >6 weeks or more (n= 776)	**Group 1:** Male: 3175 (57.0%) Female: 92 (54.1%)<br><br>**Group 2:** Male: 1661 (29.8%) Female: 39 (22.9%)<br>**Group 3:** Male: 737 (13.2%) Female: 39 (22.9%)	**Group 1: <30:** 10 (58.8%) 30-59: 2380 (58.0%) ≥60: 877 (54.0%)<br><br>**Group 2**: <30: 6 (35.3%) 30-59: 1197 (29.2%) ≥60: 497 (30.6%)<br><br>**Group 3:** <30: 1 (5.9%) 30-59: 524 (12.8%) ≥60: 251 (15.4%)	**Group 1:** Medical center: 2380 (56.4%) Local/regional: 877 (58.3%) Unknown: 10 (50.0%)<br><br>**Group 2:** Medical center: 1288 (30.5%) Local/regional: 405 (26.9%) Unknown: 7 (35.0%)<br><br>**Group 3:** Medical center: 551 (13.1%) Local/regional: 222 (14.8%) Unknown: 3 (15.0%)	NR**	**$p=0.02$**<br><br>$\chi2$ test
**HOSPITAL OWNERSHIP**						
**Categorical**						
Tsai et al., 2017 [11]	**Group 1:** <=30 days (n = 18,193)<br><br>**Group 2:** 31 to 120 days (n = 2,498)<br><br>**Group 3:** >120 days (n = 572)	**Group 1:** Male: 16,704 (85.6%) Female: 1,489 (84.7%)<br><br>**Group 2:** Male: 2,296 (11.8%) Female: 202 (11.5%)<br><br>**Group 3:** Male: 505 (2.6%) Female: 67 (3.8%)	**Group 1:** <=44: 4,304 (86.1%) 45-54: 6,337 (85.9%) 55-64: 4,296 (86.1%) ≥65: 3,257 (83.6%)<br><br>**Group 2:** <=44:567 (11.3%) 45-54: 842 (11.4%) 55-64: 554 (11.1%) ≥65: 535 (13.7%)<br><br>**Group 3:** <=44: 131 (2.6%) 45-54: 197 (2.7%) 55-64:138 (2.8%) ≥65: 106 (2.7%)	**Group 1:** Public: 4,877 (82.8%) Private: 13,316 (86.6%)<br><br>**Group 2:** Public: 850 (14.4%) Private: 1,648 (10.7%)<br><br>**Group 3:** Public: 164 (2.8%) Private: 408 (2.7%)	NR**	**$p<0.001$**<br><br>$\chi2$ test
**CARE TRANSITION/LOCATION OF INITIAL DIAGNOSIS**						
**Categorical**						
Morse et al., 2018 [24]	**Group 1: No delay** (First and second quartiles for each interval of days; n=2,012)<br><br>**Group 2: Delay** (Fourth quartile	**Group 1:** Male: 1,701 (85%) Female: 311 (15%)<br><br>**Group 2:** Male: 829 (80%)	**Group 1:** ≤60 years:1,253 (62%) >60 years: 759 (38%)<br><br>**Group 2:** ≤60 years:	**Group 1:** No care transition: 988 (49%) Care transition: 1,024 (51%)<br><br>**Group 2:** No care transition: 386 (37%)	Care transition: Ref. No care transition (OR: 1.73; 1.46 to 2.04)	Care transition: Ref.[a] No care transition: **$p< 0.001$**

	for each interval of days; n=1,032).	Female: 203 (20%)	634 (61%)<br>>60 years: 398 (39%)	Care transition: 646 (63%)		
**Continuous**						
Fujiwara et al., 2017 [12]	The fourth quartile of each treatment interval was used as an indicator of delayed treatment.<br><br>**Median=** 30 days	NR**	NR**	At reporting facility: 1,926 (39.6%)<br>Elsewhere: 2,942 (60.4%)	At reporting facility: Ref.<br>Outside facility (OR: 2.52; 2.15 to 2.95)	At reporting facility. Ref.[a]<br>Outside facility: **p<0.001**
Morse et al., 2018 [25]	Patients were classified as delayed (fourth quartile) vs not delayed (first and second quartiles) for each interval of time to treatment.<br><br>**Median=** 27 days.	NR**	NR**	No care transition: 1,536 (41%)<br>Care transition: 2,172 (59%)	No care transition: Ref.<br>Care transition (OR: 2.70; 2.1 to 3.49)	No care transition: Ref.[a]<br>Care transition: **p<0.001**
**URBANIZATION LEVEL HOSPITAL**						
**Categorical**						
Tsai et al., 2017 [11]	**Group 1:** <=30 days (n = 18,193)<br><br>**Group 2:** 31 to 120 days (n = 2,498)<br><br>**Group 3:** >120 days (n = 572)	**Group 1:**<br>Male: 16,704 (85.6%)<br>Female: 1,489 (84.7%)<br><br>**Group 2:**<br>Male: 2,296 (11.8%)<br>Female: 202 (11.5%)<br><br>**Group 3:**<br>Male: 505 (2.6%)<br>Female: 67 (3.8%)	**Group 1:**<br><=44: 4,304 (86.1%)<br>45-54: 6,337 (85.9%)<br>55-64: 4,296 (86.1%)<br>≥65: 3,257 (83.6%)<br>**Group 2:**<br><=44:567 (11.3%)<br>45-54: 842 (11.4%)<br>55-64: 554 (11.1%)<br>≥65: 535 (13.7%)<br>**Group 3:**<br><=44: 131 (2.6%)<br>45-54: 197 (2.7%)<br>55-64:138 (2.8%)<br>≥65: 106 (2.7%)	**Group 1:**<br>Level 1: 4,203 (85.5%)<br>Levels 2 & 3: 8,576 (85.5%)<br>Levels 4 & 5: 3,399 (85.6%)<br>Levels 6 & 7: 2,015 (86.1%)<br><br>**Group 2:**<br>Level 1: 587 (11.9%)<br>Levels 2 & 3: 1,178 (11.7%)<br>Levels 4 & 5: 469 (11.8%)<br>Levels 6 & 7: 264 (11.3%)<br><br>**Group 3:**<br>Level 1: 128 (2.6%)<br>Levels 2 & 3: 281 (2.8%)<br>Levels 4 & 5: 101 (2.6%)<br>Levels 6 & 7:62 (2.6%)	NR**	p=0.951<br><br>$\chi 2$ test
**DISTANCE FROM TREATMENT CENTER**						
**Categorical**						
Sharma et al., 2016 [23]	**Group 1:**<br>DTI< 30 days (n=3,020)	**Group 1:**<br>Male: 2,482 (82.2%)<br>Female: 538	**Group 1:**<br><65: 2,319 (76.8%)<br>≥65: 701	**Group 1:**<br><25 miles: 2,208 (73.1%)<br>25-100 miles: 552	<25 miles: Ref.<br>25-100 miles (OR: 1.00; 0.88 to 1.14)<br>>100 miles (OR:	<25 miles: Ref.[a]<br>25-100 miles: p>0.05<br>>100 miles: p>0.05

	**Group 2:**<br>DTI ≥ 30 days<br>(n=3,586)	(17.8%)<br><br>**Group 2:**<br>Male: 2,951<br>(82.3%)<br>Female: 635<br>(17.7%)	(23.2%)<br><br>**Group 2:**<br><65: 2,736<br>(76.3%)<br>≥65: 850<br>(23.7%)	(18.3%)<br>>100 miles: 106<br>(3.5%)<br>Unknown: 154<br>(5.1%)<br><br>**Group 2:**<br><25 miles: 2,593<br>(72.3%)<br>25-100 miles: 689<br>(19.2%)<br>>100 miles: 150<br>(4.2%)<br>Unknown: 154<br>(4.3%)	1.08; 0.83 to 1.41)<br>Unknown (OR: 0.82;<br>0.65 to 1.04)	Unknown: p>0.05
**PLACE OF RESIDENCE**						
**Continuous**						
Venchiarutti et al., 2020 [30]	NR**	NR**	NR**	**Oropharynx:**<br>Regional/remote: 47<br>(42%)<br>Metropolitan: 65<br>(58%)<br><br>**Oral cavity:**<br>Regional/remote: 37<br>(33%)<br>Metropolitan: 75<br>(66%)	**Oropharynx:**<br>Regional/ remote: 47<br>(IQR 43) median<br>days<br>Metropolitan: 36<br>(IQR 14) median<br>days<br><br>**Oral cavity:**<br>Regional/ remote: 33<br>(IQR 21) median<br>days<br>Metropolitan: 29<br>(IQR 21) median<br>days	**Oropharynx:**<br>**p=0.03**<br><br>**Oral cavity:**<br>p=0.19<br><br>(Mann–Whitney U<br>test)
Zhang et al., 2015 [31]	NR**	NR**	NR**	**Rural:** 127 (23%)<br>**Intermediate:** 140<br>(25,3%)<br>**Urban:** 287 (51,7%)	Rural:<br>53.4 (Range 4–159)<br>mean days<br>Intermediate:<br>53.3 (Range 4–152)<br>mean days<br>Urban:<br>49.3 (Range 4–155)<br>mean days.	p= 0.791<br><br>(ANOVA test).
**INCOME**						
**Categorical**						
Tsai et al., 2017 [11]	**Group 1:** <=30<br>days<br>(n = 18,193)<br><br>**Group 2:** 31 to<br>120 days<br>(n = 2,498)<br><br>**Group 3:** >120<br>days<br>(n = 572)	**Group 1:**<br>Male: 16,704<br>(85.6%)<br>Female: 1,489<br>(84.7%)<br><br>**Group 2:**<br>Male: 2,296<br>(11.8%)<br>Female: 202<br>(11.5%)<br><br>**Group 3:**<br>Male: 505<br>(2.6%)<br>Female: 67<br>(3.8%)	**Group 1:**<br><=44: 4,304<br>(86.1%)<br>45-54: 6,337<br>(85.9%)<br>55-64: 4,296<br>(86.1%)<br>≥65: 3,257<br>(83.6%)<br><br>**Group 2:**<br><=44:567<br>(11.3%)<br>45-54: 842<br>(11.4%)<br>55-64: 554<br>(11.1%)<br>≥65: 535<br>(13.7%)<br><br>**Group 3:**<br><=44: 131<br>(2.6%)<br>45-54: 197	**Group 1:**<br>Low-income: 383<br>(85.9%)<br><= 17,280: 882<br>(85.6%)<br>17,281 to 22,800:<br>9,589 (85.0%)<br>> = 22,801: 7,339<br>(86.3%)<br><br>**Group 2:**<br>Low-income: 54<br>(12.1%)<br><= 17,280: 116<br>(11.3%)<br>17,281 to 22,800:<br>1,377 (12.2%)<br>> = 22,801: 951<br>(11.2%)<br><br>**Group 3:**<br>Low-income: 9<br>(2.0%)<br><= 17,280: 32 (3.1%)	NR**	p= 0.161<br><br>$\chi2$ test

			(2.7%) 55-64:138 (2.8%) ≥65: 106 (2.7%)	17,281 to 22,800: 320 (2.8%) > = 22,801: 211 (2.5%)		
**Continuous**						
Goel et al., 2019 [13]	Time to treatment was presented as a continuous variable (number of days).<br><br>**Median DTI=** 26 days	NR**	NR**	<38,000: 433 (12.2%) 38,000-47,999: 706 (19.9%) 48,000-62,999: 1,000 (28.2%) ≥63,000: 1,401 (39.5%)	<38.000: Ref. 38.000-47.999 (Coefficient: −0.11; −2.87 to 2.64) 48.000-62.999 (Coefficient: 0.78; −1.99 to 3.56) ≥63.000 (Coefficient: 0.55; −2.45 to 3.55)	<38.000: Ref.[a] 38.000-47.999: p=0.94 48.000-62.999: p=0.58 ≥63.000: p=0.72
Rogers et al., 2007 [29]	The time between diagnosis and treatment was presented as a continuous variable (median days).<br><br>**Median=** 21 days (IQR 12-30).	NR**	NR**	IMD 2000 Deprivation tertile groups<br><br>Median rank 149= 175 (31,3%) Median rank 1308= 175 (31,3%) Median rank 4642= 177 (37,4%)	**Median days:** Median rank 149= 20 days (IQR 12–29) Median rank 1308= 21 days (IQR 13–30) Median rank 4642= 21 days (IQR 12–32)	p=0.50<br><br>(Kruskal–Wallis test).
**EDUCATION**						
**Continuous**						
Goel et al., 2019 [13]	Time to treatment was presented as a continuous variable (number of days).<br><br>**Median DTI=** 26 days (IQR, 14-39 days).	NR**	NR**	≥21%: 377 (10.6%) 13%-20.9%: 821 (23.1%) 7%-12.9%: 1,257 (35.4%) <7%: 1089 (30.7%)	≥21%: Ref. 13%-20.9% (Coefficient: −2.01; −4.77 to 0.75) 7%-12.9% (Coefficient: −3.12; −6.04 to −0.21) <7% (Coefficient: -6.42; −9.61 to −3.22)	≥21%: Ref.[a] 13%-20.9%: p=0.15 7%-12.9%: **p=0.04** <7%: **p<0.001**
**RACE**						
**Categorical**						
Sharma et al., 2016 [23]	**Group 1:** DTI< 30 days (n=3,020) **Group 2:** DTI ≥ 30 days (n=3,586)	**Group 1:** Male: 2,482 (82.2%) Female: 538 (17.8%)<br><br>**Group 2:** Male: 2,951 (82.3%) Female: 635 (17.7%)	**Group 1:** <65: 2,319 (76.8%) ≥65: 701 (23.2%)<br><br>**Group 2:** <65: 2,736 (76.3%) ≥65: 850 (23.7%)	**Group1:** Non-Hispanic White: 2,594 (85.9%) Non-Hispanic Black: 284 (9.4%) Hispanic: 70 (2.4%) Other/unknown: 70 (2.4%)<br><br>**Group 2:** Non-Hispanic White: 2,906 (81.0%) Non-Hispanic Black: 449 (12.5%) Hispanic: 155 (4.3%) Other/unknown: 76 (2.1%)	Non-Hispanic White: Ref Non-Hispanic Black (OR: 1.24; 1.04 to 1.46) Hispanic (OR: 1.74; 1.29 to 2.34) Other/unknown (OR: 0.84; 0.60 to 1.17)	Non-Hispanic White: Ref.[a] Non-Hispanic Black: **p< 0.05** Hispanic: **p<0.001** Other/unknown: p>0.05
Morse et al. 2018 [24]	**Group 1: No delay** (First and second quartiles for each interval of days;	**Group 1:** Male: 1,701 (85%) Female: 311 (15%)	**Group 1:** ≤60 years:1,253 (62%) >60 years:	**Group 1:** Non-White: 212 (11%) White: 1,770 (88%) Unknown: 30 (1%)	Non-White: Ref White (OR: 0.69; 0.54 to 0.88) Unknown (OR: 0.76; 0.39 to 1.50)	Non-White: Ref.[a] White: **p= 0.003** Unknown: p=0.428

	n=2,012)<br>**Group 2: Delay** (Fourth quartile for each interval of days; n=1,032)	**Group 2:** Male: 829 (80%) Female: 203 (20%)	759 (38%)<br><br>**Group 2:** ≤60 years: 634 (61%) >60 years: 398 (39%)	**Group 2:** Non-White: 179 (17%) White: 836 (81%) Unknown: 17 (2%)		
**Continuous**						
Fujiwara et al., 2017 [12]	The fourth quartile of each treatment interval was used as an indicator of delayed treatment.<br><br>**Median**= 30 days.	NR**	NR**	White: 4,304 (88.4%) Non-White: 564 (11.6%)	Non-White: Ref. White: (OR: 0.96; 0.77 to 1.21)	Non-White: Ref.[a] White: p=0.75
Morse et al., 2018 [25]	Patients were classified as delayed (fourth quartile) vs not delayed (first and second quartiles) for each interval of time to treatment.<br><br>**Median**= 27 days.	NR**	NR**	Nonwhite: 292 (8%) White: 3,356 (91%) Unknown: 60 (2%)	Non-white: Ref. White (OR: 0.88; 0.59 to 1.31) Unknown (OR: 1.27; 0.53 to 3.03)	Non-white: Ref.[a] White: p= 0.524 Unknown: p=0.595
Goel et al., 2019 [13]	Time to treatment was presented as a continuous variable (number of days).<br><br>**Median**= 26 days	NR**	NR**	White: 3,342 (94.1%) Black: 134 (3.8%) Other: 74 (2.1%)	White: Ref. Black (Coefficient: 1.44; −2.27 to 5.16) Other (Coefficient: −1.21; −6.03 to 3.61)	White: Ref.[a] Black: p=0.45 Other: p=0.62
**MARITAL STATUS**						
**Continuous**						
Rogers et al., 2007 [29]	The time between diagnosis and treatment was presented as a continuous variable (median days).<br><br>**Median**= 21 days (IQR 12-30).	NR**	NR	Married= 265 (53,3%) Single= 76 (15,3%) Divorced= 66 (13,3%) Widowed= 90 (18,1%)	**Median days:** Married: 20 days (IQR 12-29) Single: 21 days (IQR 14-34) Divorced: 22 days (IQR 13-35) Widowed= 21 (IQR 13-31)	p=0.62<br><br>(Kruskal–Wallis test).

[a]Ref.: Reference.

Box S4 Methodological quality using Joanna Briggs Appraisal tool for cohort studies. Low methodological quality is represented in red (no); unclear is represented in yellow; high quality is represented in green (yes). "Not applicable" (in blue) was used for articles where data loss was an exclusion criterion.

**Study**	**1**	**2**	**3**	**4**	**5**	**6**	**7**	**8**	**9**	**10**	**11**
Sharma et al., 2016 [25]	Yes	Yes	Yes	Yes	Yes	Yes	Yes	Yes	Not applicable	Not applicable	Yes
Morse et al., 2018(a) [26]	Yes	Yes	Yes	No	No	Yes	Yes	Yes	Yes	No	Yes
Su et al., 2021 [29]	Yes	Yes	Yes	No	No	Yes	Yes	Yes	Not applicable	Not applicable	Yes
Tsai et al., 2017 [11]	Yes	Yes	Yes	No	No	Yes	Yes	Yes	Yes	No	Yes
Fujiwara et al., 2017 [12]	Yes	Yes	Unclear	No	No	Yes	Yes	Yes	Not applicable	Not applicable	Yes
Morse et al., 2018 (b) [27]	Yes	Yes	Unclear	No	No	Yes	Yes	Yes	Not applicable	Not applicable	Yes
Venchiarutti et al., 2020 [30]	Yes	Yes	Yes	No	No	Yes	Yes	Yes	Not applicable	Not applicable	Yes
Zhang et al., 2015 [31]	Yes	Yes	Yes	No	No	Yes	Yes	Yes	Not applicable	Not applicable	Yes
Rogers et al., 2007 [28]	Yes	Yes	Yes	No	No	Yes	Yes	Yes	Yes	Yes	Yes
Goel et al., 2019 [13]	Yes	Yes	Yes	No	No	Yes	Yes	Yes	Yes	No	Yes

1. Were the two groups similar and recruited from the same population? 2. Were the exposures measured similarly to assign people to both exposed and unexposed groups? 3. Was the exposure measured in a valid and reliable way? 4. Were confounding factors identified? 5. Were strategies to deal with confounding factors stated? 6. Were the groups/participants free of the outcome at the start of the study (or at the moment of exposure)? 7. Were the outcomes measured in a valid and reliable way? 8. Was the follow up time reported and sufficient to be long enough for outcomes to occur? 9. Was follow up complete, and if not, were the reasons to loss to follow up described and explored? 10. Were strategies to address incomplete follow up utilized? 11. Was appropriate statistical analysis used
